# Sleep and wake cycles dynamically modulate hippocampal inhibitory synaptic plasticity

**DOI:** 10.1371/journal.pbio.3001812

**Published:** 2022-11-01

**Authors:** Kunwei Wu, Wenyan Han, Wei Lu

**Affiliations:** Synapse and Neural Circuit Research Section, National Institute of Neurological Disorders and Stroke, National Institutes of Health, Bethesda, Maryland, United States of America; Columbia University Irving Medical Center, UNITED STATES

## Abstract

Sleep is an essential process that consolidates memories by modulating synapses through poorly understood mechanisms. Here, we report that GABAergic synapses in hippocampal CA1 pyramidal neurons undergo daily rhythmic alterations. Specifically, wake inhibits phasic inhibition, whereas it promotes tonic inhibition compared to sleep. We further utilize a model of chemically induced inhibitory long-term potentiation (iLTP) to examine inhibitory plasticity. Intriguingly, while CA1 pyramidal neurons in both wake and sleep mice undergo iLTP, wake mice have a much higher magnitude. We also employ optogenetics and observe that inhibitory inputs from parvalbumin-, but not somatostatin-, expressing interneurons contribute to dynamic iLTP during sleep and wake. Finally, we demonstrate that synaptic insertion of α5-GABA_A_ receptors underlies the wake-specific enhancement of iLTP at parvalbumin-synapses, which is independent of time of the day. These data reveal a previously unappreciated daily oscillation of inhibitory LTP in hippocampal neurons and uncover a dynamic contribution of inhibitory synapses in memory mechanisms across sleep and wake.

## Abstract 2

An electrophysiological study reveals a previously unappreciated daily oscillation in inhibitory synaptic plasticity and uncovers a dynamic contribution of inhibitory synapses to memory mechanisms across both sleep and wake states.

## Introduction

Sleep is an essential and widely expressed behavior across the animal kingdom. In addition to the numerous health benefits that are connected to sleep, there is a large body of literature that contributes sleep to mental processes such as learning and memory. It has been demonstrated that sleep is critical for memory consolidation and that sleep deprivation perturbs memory processes, likely through the modulation of synapses [1–4]. Recently, the impact of sleep on synaptic strength has been extensively studied at excitatory synapses [5–8]. Indeed, several studies have revealed a decreased abundance of synaptic AMPA-type glutamate receptors [9,10] and excitatory synaptic strength [11–13], and spine density in association with sleep [11,14,15]. Sleep has also been shown to promote spine elimination [68]. In addition, reduced excitatory transmission during sleep may facilitate the induction of long-term potentiation (LTP) [10]. However, other studies have shown that sleep could potentiate excitatory synaptic transmission [16], have no impact on excitatory synaptic strength [17], or promote spine formation after learning [69], indicating that synaptic mechanisms regulated by sleep/wake states are likely complex and warrant additional work to fully evaluate the role of sleep in regulating excitatory synaptic transmission.

In contrast to extensive studies on modulation of excitatory synapses during the sleep and wake cycle [5–8], much less is known about how inhibitory transmission is regulated by sleep. As synaptic excitation and inhibition are balanced to stabilize neuronal and circuit function and modulation of inhibitory transmission plays a critical role in learning and memory [18–20], it is critical to understand the impact of sleep on inhibitory transmission. In the mammalian brain, inhibitory transmission is principally mediated by GABA acting on GABA_A_ receptors (GABA_A_Rs) [21]. The basal inhibitory transmission includes phasic inhibition mediated by synaptic GABA_A_Rs and tonic inhibition mediated by extrasynaptic GABA_A_Rs [22]. It has been reported that a brief slow-wave sleep or arousal state (approximately 15 to 20 min) is sufficient to increase or decrease phasic inhibition in rat cortical neurons, respectively, through modulation of GABA_A_R trafficking [23]. Recently, it has also been shown that GABA_A_R-mediated tonic inhibition alters over the sleep and wake cycle [24], and synaptic inhibition changes at different times of the day [25,26], indicating that GABAergic inhibition is dynamically regulated by the sleep and wake cycle as well as circadian rhythm.

Here, we have employed a real-time, non-invasive sleep tracking system to monitor sleep/wake states in mice and recorded GABAergic inhibitory transmission in hippocampal CA1 neurons under basal conditions and during synaptic plasticity. We have discovered sleep-dependent dynamics of GABAergic inhibition, identified an input-specific change of GABAergic inhibitory LTP (iLTP) across sleep and wake states, and revealed a critical role of synaptic insertion of α5-GABA_A_Rs in iLTP in a wake-dependent manner.

## Results

### GABAergic transmission changes across sleep and wake states

**Fig 1:**
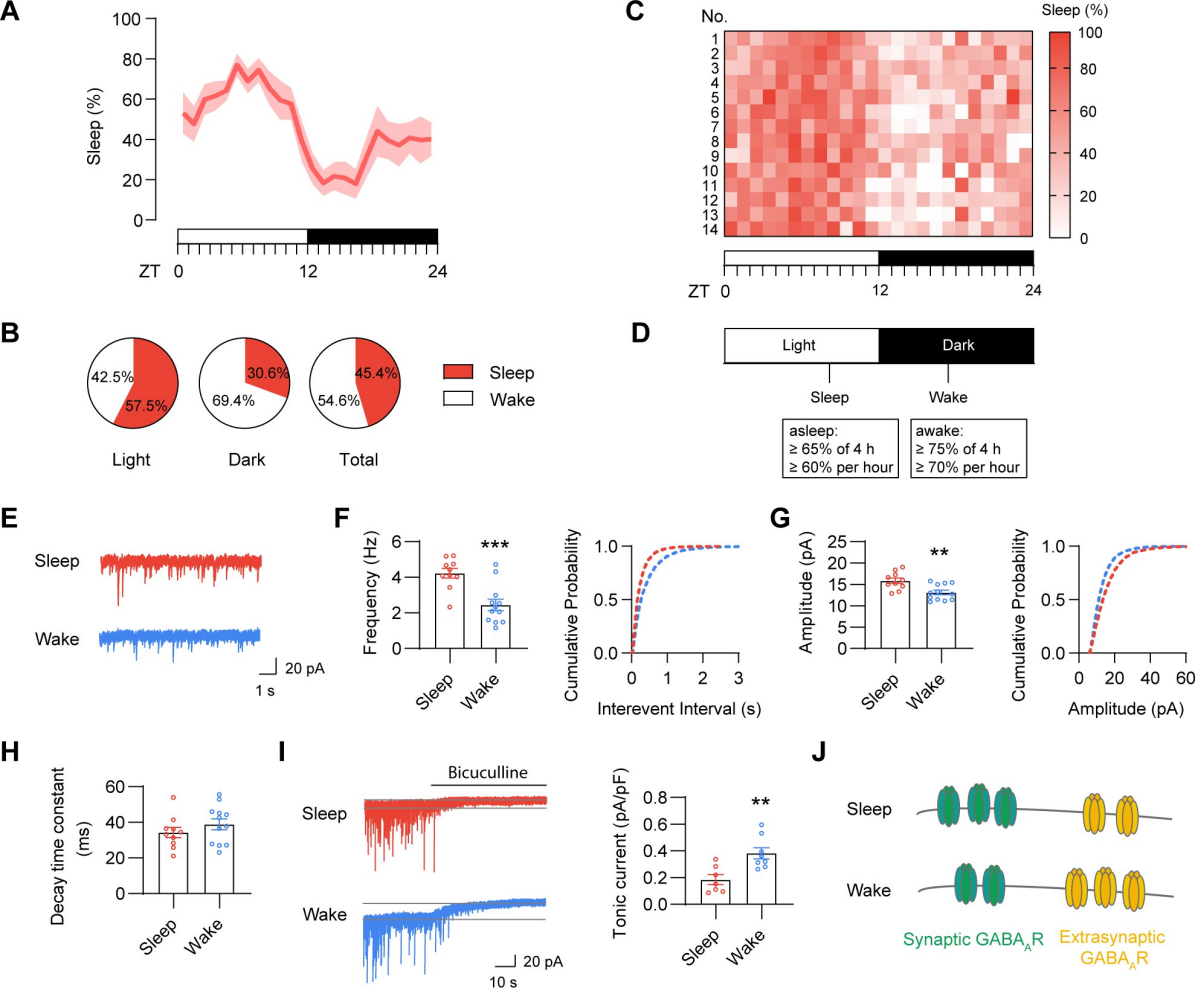
GABAergic transmission and GABA_A_R expression change across sleep and wake. ** (A) Sleep pattern over 24 h in wild-type 8–12 weeks old male mice (*n* = 14).** (B) Percentage of sleep time spent in light phase, dark phase, and the whole day (*n* = 14). (C) Percentages of hourly sleep time were plotted over 24 h (*n* = 14). (D) Definition of sleep/wake mice: “sleep mice” were asleep for at least 65% of the previous 4 h (≥60% per hour), whereas “wake mice” were awake for at least 75% of the previous 4 h (≥70% per hour). Mice were selected for electrophysiological or biochemical experiments only if they met the criteria. (E) Representative mIPSC traces from CA1 neurons in acute hippocampal slices prepared from sleep and wake mice. (F, G) mIPSC frequency and amplitude were decreased in hippocampal CA1 pyramidal neurons in wake mice compared to sleep mice (*n* = 10–12, *t* test, frequency: *p* = 0.0005, amplitude: *p* = 0.0036). (H) There was no difference of mIPSC decay time constants in sleep and wake mice (*n* = 10–12, *t* test). (I) Tonic inhibition was increased in hippocampal CA1 pyramidal neurons in wake mice compared to sleep mice (*n* = 7–8, *t* test, *p* = 0.0043). (J) A model showing the changes of synaptic and extrasynaptic GABA_A_R expression across sleep and wake. The data underlying this figure can be found in S1 Data. ***p < 0.001 and **p < 0.01. All data are presented as mean ± SEM. mIPSC, miniature inhibitory postsynaptic current.

In order to assess whether sleep/wake induces changes of GABAergic inhibition, we employed a PiezoSleep mouse behavioral tracking system to monitor sleep/wake states in young adult mice (8 to 12 weeks old). Based on the sleep–wake pattern recorded by the PiezoSleep system (Fig 1A–1C) and the criteria used in previous studies [11,24], we defined “sleep” and “wake” mice with the following criteria: “sleep mice” were asleep for at least 65% of the previous 4 h (≥60% per hour), whereas “wake mice” were awake for at least 75% of the previous 4 h (≥70% per hour) (Fig 1D). Mice were selected for electrophysiology experiments only if they met the criteria. Each individual mouse has their own sleep–wake pattern and they may be collected at different times of day based on our sleep/wake definition (Fig 1C and 1D). Sleep mice were sacrificed during the light phase (ZT6-9) and wake mice were sacrificed during the dark phase (ZT16-19). As hippocampal CA1 area is a critical brain region for diverse functions including memory consolidation during sleep [27], we thus focused on GABAergic transmission in hippocampal CA1 neurons in this study. Following sacrifice and preparation of acute hippocampal slices, CA1 pyramidal neurons were recorded using patch clamp. We found that wake inhibited the amplitude and frequency of miniature inhibitory postsynaptic currents (mIPSCs) without affecting mIPSC decay (Fig 1E–1H). In contrast, in agreement with our previous report [24], tonic inhibitory currents were increased in wake mice compared to sleep mice (Fig 1I). GABA_A_R expression in the plasma membrane and at synaptic sites is a critical determinant of the strength of GABAergic inhibition [28]. Generally, GABA_A_Rs can be classified as mediating either phasic or tonic inhibition. Specifically, in the hippocampus, phasic inhibition is primarily mediated by synaptically localized α1/α2-GABA_A_Rs that respond to presynaptic GABA release, whereas tonic inhibition is mainly mediated by α4/α5-GABA_A_Rs localized either extrasynaptically or perisynaptically that respond to low ambient levels of GABA [22,29]. By analyzing the GABA_A_R expression in hippocampal homogenates and crude synaptosomes, we found that while there was no change in GABA_A_R subunits from total homogenates across sleep and wake states, wake decreased α1/α2-GABA_A_Rs expression but increased α4/α5-GABA_A_Rs expression in the crude synaptosomes (S1A–S1D Fig), consistent with the electrophysiological data (Fig 1E–1I). Given that some α4/α5-GABA_A_Rs may localize out of the crude synaptosomes, we further confirmed the increase of α4/α5-GABA_A_R surface expression in the hippocampi in wake mice using a surface biotinylation assay (S1E and S1G Fig). Previous work has shown that the tonic inhibition in hippocampal CA1 neurons is mainly mediated by α5-GABA_A_Rs [30,31]. To further determine whether the α5-GABA_A_Rs contributed to the increase of tonic inhibition in CA1 pyramidal neurons in wake state, we applied a potent α5-GABA_A_R inverse agonist, L655,708 and found that wake increased the L655,708-sensitive tonic currents without altering the L-655,708-insensitive tonic currents, highlighting that the wake-induced increase in tonic inhibition is mediated by α5-GABA_A_Rs (S1H–S1J Fig). Taken together, these results provide biochemical and electrophysiological evidence supporting dynamic changes in GABAergic transmission and GABA_A_R expression across sleep and wake states (Fig 1J).

Given that the sleep and wake mice were sacrificed at different times of day, it was important to explore whether the observed electrophysiological changes were induced by wake/sleep states or time of the day. For this purpose, 1 group of mice were sleep-deprived for 4 h during the light phase, when they would normally have slept. These sleep-deprived mice were then sacrificed for electrophysiological recordings at the same time with the control group, normally sleep mice. Similar to wake mice, sleep-deprived mice exhibited a decrease in mIPSC frequency and amplitude as well as an increase in tonic inhibition in hippocampal CA1 pyramidal neurons without affecting mIPSC decay (S2 Fig). These results indicate that sleep-/wake-dependent difference in GABAergic transmission is independent of time of the day, instead depending on the sleep/wake history of the mice.

### Enhancement of iLTP by the wakefulness

**Fig 2:**
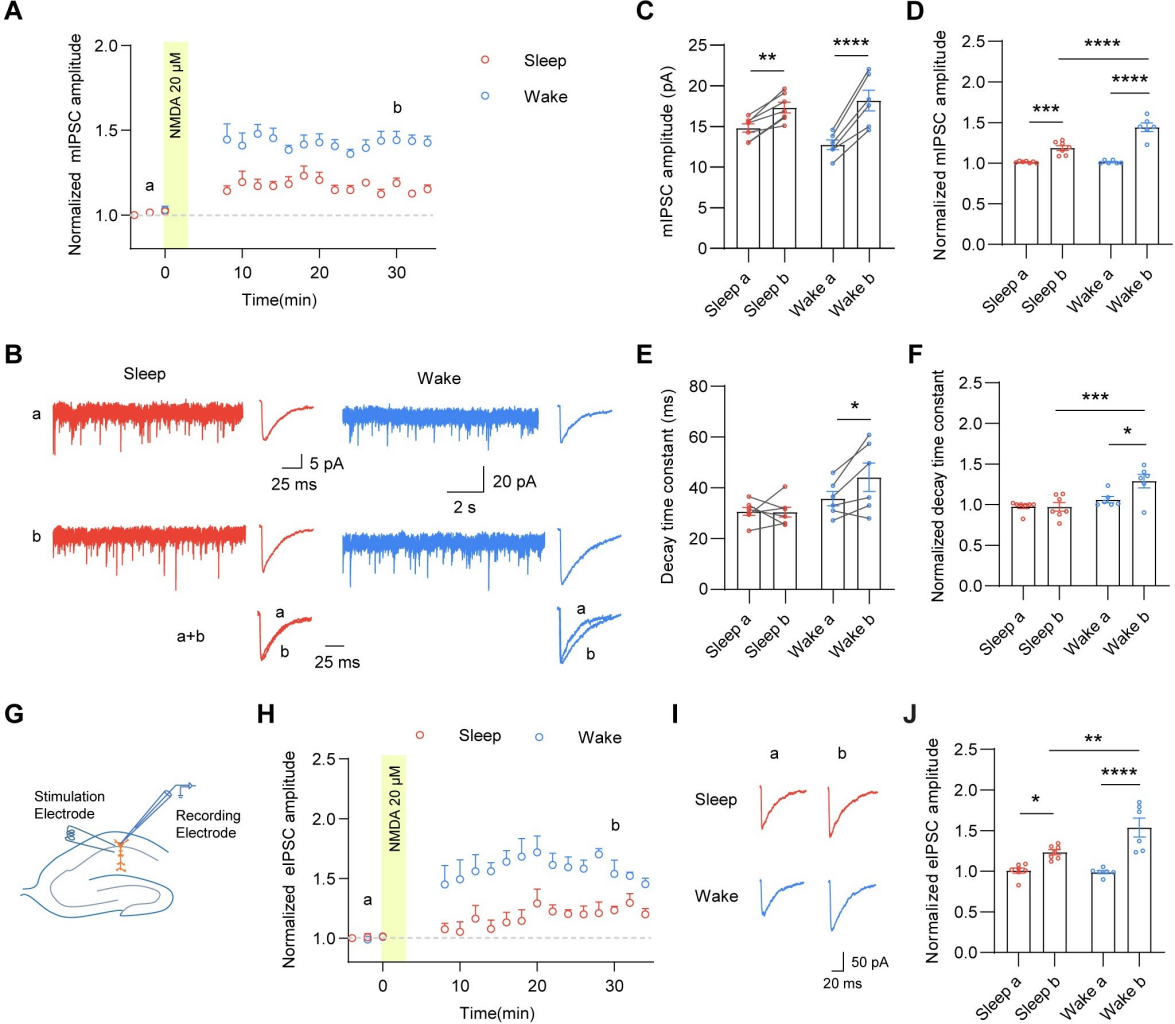
Enhancement of iLTP by the wakefulness. (A) Brief NMDA exposure (3 min, 20 μM) to hippocampal slices was sufficient to induce a persistent increase in mIPSC amplitude over an approximately 30-min period in sleep and wake mice. The data were binned into 2-min time bins. (B) Representative mIPSC traces and average mIPSC traces at indicated time points in (A) from the CA1 pyramidal neuron in acute hippocampal slices prepared from sleep and wake mice. a+b indicated peak-scaled overlays showing the difference of decay time constants between time points a and b. (C) NMDA application induced a persistent increase in mIPSC amplitude in CA1 pyramidal neurons in wake and sleep mice (*n* = 6–7, 2-way ANOVA, F (1, 11) = 89.98, *p* < 0.0001 with Sidak’s multiple comparison test, Sleep a versus Sleep b, *p* = 0.0021; Wake a versus Wake b, *p* < 0.0001). (D) mIPSC amplitude in CA1 pyramidal neurons in wake mice 30 min post-NMDA application had a higher potentiation than sleep mice (*n* = 6–7, 2-way ANOVA, F (1, 22) = 19.11, *p* = 0.0002 with Sidak’s multiple comparison test, Sleep a versus Sleep b, *p* = 0.0004; Wake a versus Wake b, *p* < 0.0001; Sleep b versus Wake b, *p* < 0.0001). (E, F) NMDA application enhanced mIPSC decay time constant in wake mice but not sleep mice (E: *n* = 6–7, 2-way ANOVA, F (1, 22) = 4.572, *p* = 0.043 with Sidak’s multiple comparison test, Sleep b versus Wake b, *p* = 0.014. F: *n* = 6–7, 2-way ANOVA, F (1, 22) = 4.918, *p* = 0.0372 with Sidak’s multiple comparison test, Sleep b versus Wake b, *p* = 0.014; Wake a versus Wake b, *p* = 0.0006). (G) IPSCs evoked by electrical stimulation (eIPSCs) in perisomatic region. (H) Time course of eIPSC amplitude before and after NMDA application. (I) Representative eIPSC traces at indicated time point in (H) from CA1 pyramidal neurons in acute hippocampal slices prepared from sleep and wake mice. (J) eIPSC amplitude in CA1 pyramidal neurons in wake mice 30 min post-NMDA application had a higher potentiation than sleep mice (*n* = 6–7, 2-way ANOVA, F (1, 22) = 7.403, *p* = 0.0125 with Sidak’s multiple comparison test, Sleep a versus Sleep b, *p* = 0.02; Wake a versus Wake b, *p* < 0.0001; Sleep b versus Wake b, *p* = 0.003). The data underlying this figure can be found in S1 Data. *p < 0.05, **p < 0.01, ***p < 0.001, and ****p < 0.0001. All data are presented as mean ± SEM. iLTP, inhibitory long-term potentiation; mIPSC, miniature inhibitory postsynaptic current.

Accumulating evidence has shown that GABAergic synapses are highly plastic, exhibiting activity-dependent and long-term changes in synaptic efficacy [32–34]. While most studies on inhibitory plasticity have focused on mechanistic understandings such as induction requirements, expression and maintenance mechanisms [32,35], much less is known about how inhibitory plasticity is regulated by behavioral states such as sleep. Here, we adopted a chemical protocol to induce inhibitory long-term potentiation (iLTP) and assessed whether iLTP was altered in hippocampal neurons across sleep and wake states. Specifically, following transient application of NMDA (3 min, 20 μM) in the bath perfusate, we examined mIPSC amplitude in a time-dependent manner up to 30 min after NMDA exposure in CA1 pyramidal neurons from acute hippocampal slices. In agreement with previous studies [36–38], brief NMDA exposure to hippocampal neurons was sufficient to induce a persistent increase in mIPSC amplitude (Fig 2A–2D). Similarly, NMDA also potentiated electrically evoked IPSCs (eIPSCs) in the perisomatic region in both sleep and wake mice (Fig 2G–2J). Strikingly, mIPSC/eIPSC amplitude in CA1 pyramidal neurons in wake mice 30 min post-NMDA application had a higher potentiation than that in sleep mice (Fig 2D and 2J), showing a wake-specific enhancement of iLTP. These findings suggest that sleep/wake states not only impact basal inhibitory synaptic strength, but also regulate inhibitory synaptic plasticity.

### Synaptic recruitment of α5-GABA_A_Rs contributes to wake-dependent enhancement of iLTP

**Fig 3:**
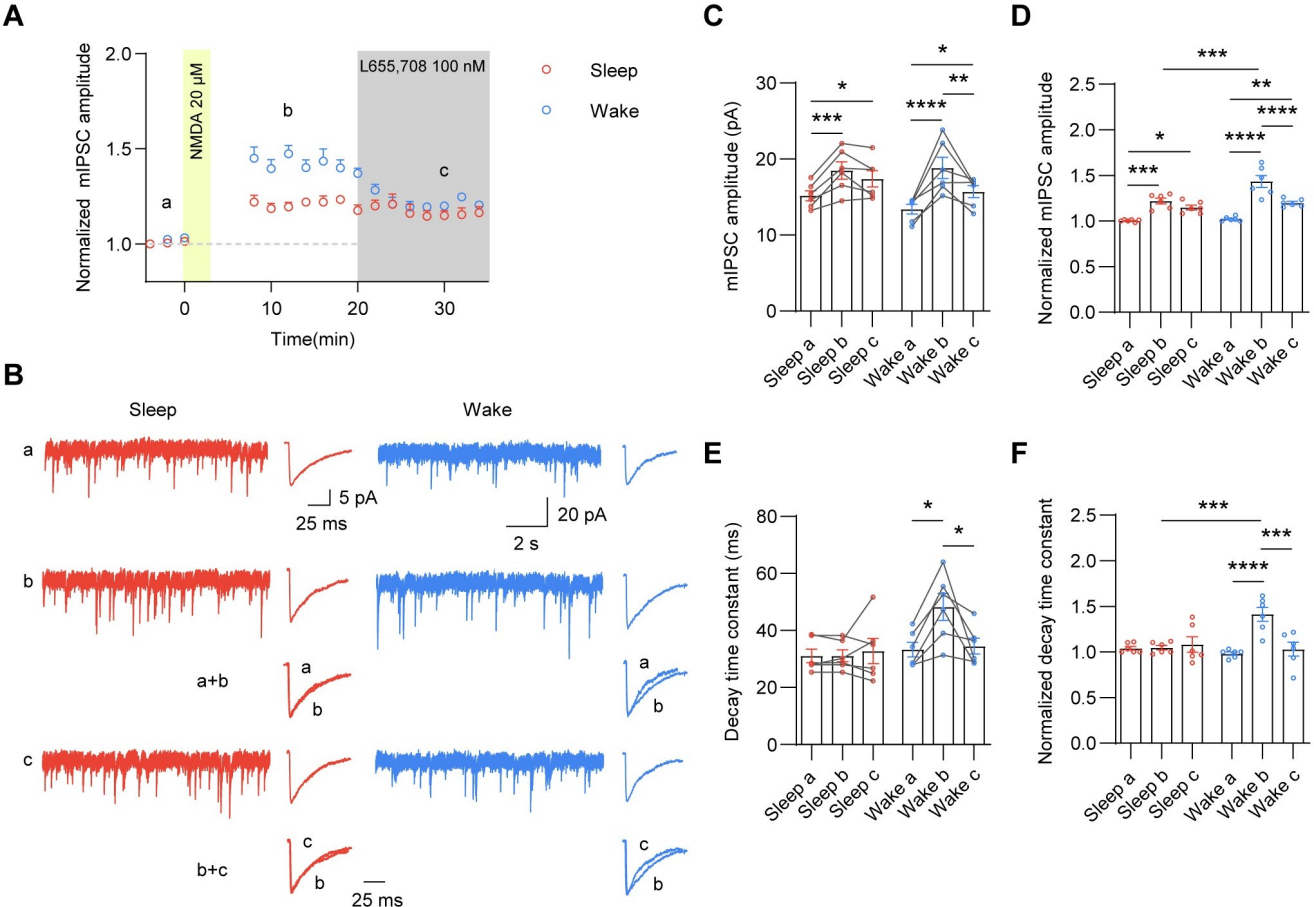
Synaptic recruitment of α5-GABA_A_Rs contributes to wake-dependent enhancement of iLTP. (A) Time course of mIPSC amplitude in hippocampal CA1 pyramidal cells before and after NMDA application. α5-GABA_A_R inverse agonist L655,708 was applied 20 min post-NMDA application. The data were binned into 2-min time bins. (B) Representative mIPSC traces and average mIPSC traces at indicated time points in (A) from the CA1 pyramidal neuron in acute hippocampal slices prepared from sleep and wake mice. a+b indicated peak-scaled overlays showing the difference of decay time constants between time points a and b. b+c indicated peak-scaled overlays showing the difference of decay time constants between time points b and c. (C) L655,708 decreased mIPSC amplitude after NMDA application in wake mice but not sleep mice. (*n* = 6, 2-way ANOVA, F (2, 20) = 34.77, *p* < 0.0001 with Sidak’s multiple comparison test, Sleep a versus Sleep b, *p* = 0.0007; Sleep a versus Sleep c, *p* = 0.0213; Wake a versus Wake b, *p* < 0.0001; Wake b versus Wake c, *p* = 0.0164; Wake a versus Wake c, *p* = 0.0013). (D) L655,708 decreased the potentiation of mIPSC amplitude after NMDA application in wake mice but not sleep mice. (*n* = 6, 2-way ANOVA, F (2, 30) = 5.215, *p* = 0.0114 with Sidak’s multiple comparison test, Sleep a versus Sleep b, *p* = 0.0003; Sleep a versus Sleep c, *p* = 0.0237; Wake a versus Wake b, *p* < 0.0001; Wake b versus Wake c, *p* < 0.0001; Wake a versus Wake c, *p* = 0.037; Sleep b versus Wake b, *p* = 0.0002). (E, F) NMDA application enhanced mIPSC decay time constant in wake mice but not sleep mice. The wake-specific enhancement of mIPSC decay time constant was abolished by L655,708 (E: *n* = 6, 2-way ANOVA, F (2, 15) = 2.972, *p* = 0.0818 with Sidak’s multiple comparison test, Wake a versus Wake b, *p* = 0.02; Wake b versus Wake c, *p* = 0.038. F: *n* = 6, 2-way ANOVA, F (2, 15) = 9.892, *p* = 0.0018 with Sidak’s multiple comparison test, Wake a versus Wake b, *p* < 0.0001; Wake b versus Wake c, *p* < 0.0004; Sleep b versus Wake b, *p* = 0.0003). The data underlying this figure can be found in S1 Data. *p < 0.05, **p < 0.01, ***p < 0.001, and ****p < 0.0001. All data are presented as mean ± SEM. iLTP, inhibitory long-term potentiation; mIPSC, miniature inhibitory postsynaptic current.

To investigate the mechanism underlying wake-dependent enhancement of iLTP, we first determined whether iLTP was induced by a postsynaptic mechanism in sleep/wake states. To this end, we applied BAPTA (10 mM), a fast Ca^2+^ chelator through the recording pipette and found that it prevented the potentiation in both sleep and wake states (S3A–S3C Fig), suggesting a postsynaptic mechanism dependent on an intracellular Ca^2+^ rise. Next, we examined NMDAR expression across sleep and wake states and found that there was no change in surface and total NMDARs (S3D–S3F Fig), indicating that the alteration of NMDA-induced iLTP across sleep and wake states is not due to differential NMDAR expression. Interestingly, we also observed that mIPSC decay time constant was significantly enhanced 30 min post-NMDA application in wake but not sleep mice (Fig 2E and 2F), suggesting that GABA_A_R subunit composition may be altered during iLTP in wake mice. GABA_A_Rs with distinct subunit composition and properties are present at both synaptic and extrasynaptic membranes [39]. Thus, it is possible that extrasynaptic receptors were recruited into synapses during iLTP in wake states, altering the decay kinetics of mIPSCs. In hippocampal CA1 pyramidal neurons, it has been reported that α5-GABA_A_Rs mediate a slowly decaying component of GABAergic inhibition [40–43]. Additionally, we showed that hippocampal α5-GABA_A_R surface expression and α5-GABA_A_R-mediated tonic currents in CA1 pyramidal neurons were increased in wake states (S1 Fig). Therefore, we speculated that α5-GABA_A_Rs might be incorporated into inhibitory synapses during iLTP in wake states. To test this, we perfused α5-GABA_A_R inverse agonist L655,708 20 min post-NMDA application and found that the wake-specific enhancement of mIPSC amplitude as well as the decay time constant were abolished (Fig 3), indicating that iLTP is strengthened in wake due to synaptic recruitment of α5-GABA_A_Rs. In contrast, L655,708 did not affect the potentiation in sleep mice, suggesting that α5-GABA_A_Rs contribute to iLTP in wake but not sleep states.

### Recruitment of α5-GABA_A_Rs to PV-synapses contributes to wake-dependent enhancement of iLTP

**Fig 4:**
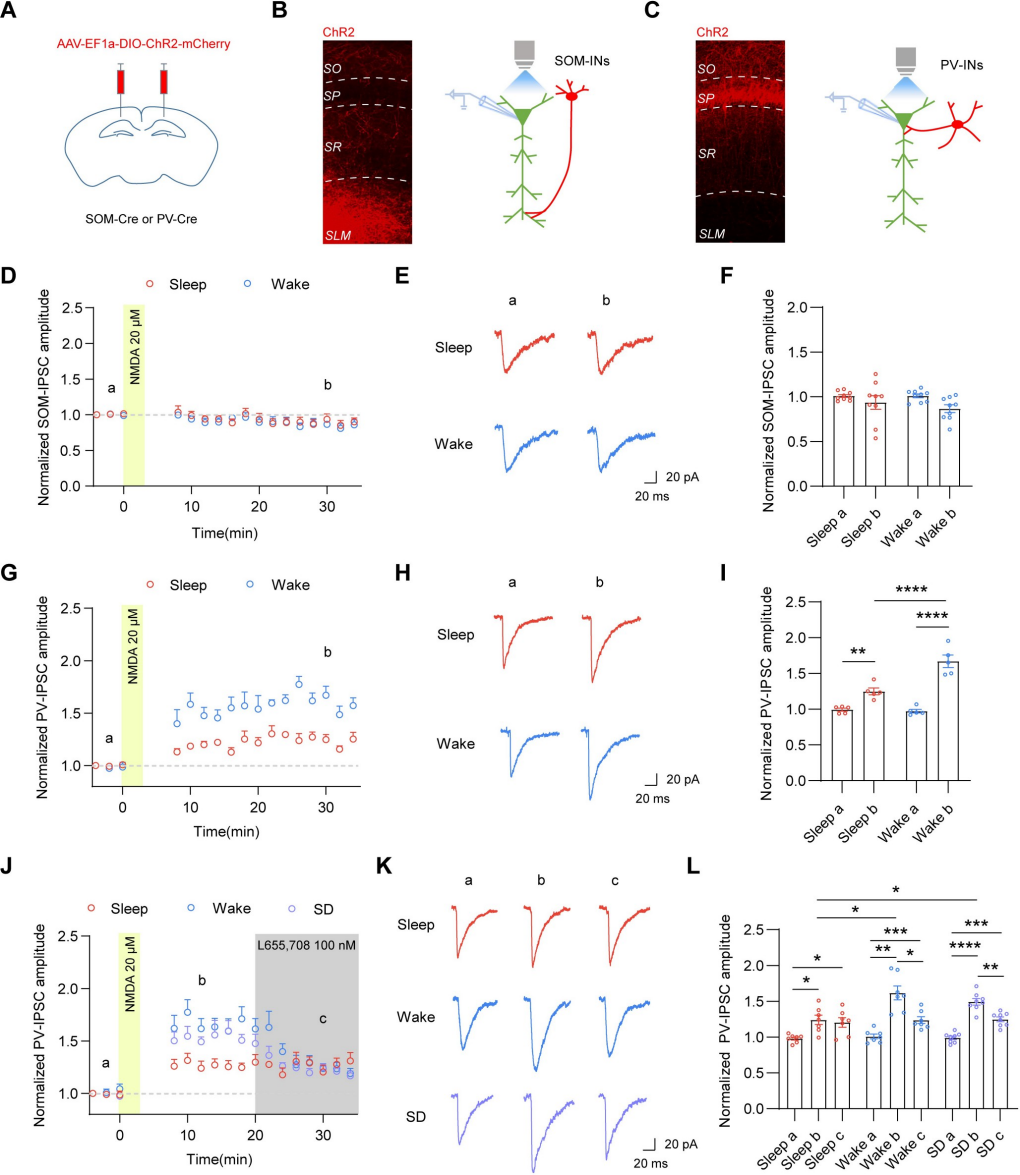
Recruitment of α5-GABA_A_Rs to PV-synapses contributes to wake-dependent enhancement of iLTP. (A) Scheme of the injection of AAV-EF1a-DIO-ChR2-mCherry into the hippocampal CA1 region of SOM-IRES-Cre or PV-IRES-Cre mice. (B, C) Left: Fluorescence image showing ChR2 expression in different hippocampal layers of SOM-IRES-Cre (B) or PV-IRES-Cre mice (C). SO, stratum oriens; SP, stratum pyramidale; SR, stratum radiatum; SLM, stratum lacunosum-moleculare. Right: Schematic displaying the recording set up for iLTP experiments. (D) Time course of IPSCs in hippocampal CA1 pyramidal cells evoked by photo-activation of SOM-INs (SOM-IPSCs) before and after NMDA application. The data were binned into 2-min time bins. (E) Representative SOM-IPSC traces at indicated time points in (D). (F) There were no changes of SOM-IPSC amplitude before and after NMDA application in sleep and wake mice (*n* = 9, 2-way ANOVA with Sidak’s multiple comparison test). (G) Time course of IPSCs in hippocampal CA1 pyramidal cells evoked by photo-activation of PV-INs (PV-IPSCs) before and after NMDA application. The data were binned into 2-min time bins. (H) Representative PV-IPSC traces at indicated time points in (G). (I) PV-IPSCs underwent iLTP in sleep and wake mice. PV-iLTP had a higher magnitude in wake mice compared to sleep mice (*n* = 5, 2-way ANOVA, F (1, 8) = 40.32, *p* = 0.0002 with Sidak’s multiple comparison test, Sleep a versus Sleep b, *p* = 0.0064; Sleep b versus Wake b, *p* < 0.0001; Wake a versus Wake b, *p* < 0.0001). (J) Time course of PV-IPSCs in hippocampal CA1 pyramidal cells before and after NMDA application. L655,708 was applied 20 min post-NMDA application. The data were binned into 2-min time bins. (K) Representative PV-IPSC traces at indicated time points in (J). (L) L655,708 decreased the potentiation of PV-IPSC amplitude after NMDA application in wake and sleep-deprived mice (SD) but not sleep mice (*n* = 7–8, 2-way ANOVA, F (4, 38) = 4.234, *p* = 0.0062 with Sidak’s multiple comparison test, Sleep a versus Sleep b, *p* = 0.028; Sleep a versus Sleep c, *p* = 0.04; Wake a versus Wake b, *p* = 0.0014; Wake b versus Wake c, *p* = 0.023; Wake a versus Wake c, *p* = 0.0008; SD a versus SD b, *p* < 0.0001; SD b versus SD c, *p* = 0.0091; SD a versus SD c, *p* = 0.0002; Sleep b versus Wake b, *p* = 0.028; Sleep b versus SD b, *p* = 0.04). The data underlying this figure can be found in S1 Data. *p < 0.05, **p < 0.01, ***p < 0.001, and ****p < 0.0001. All data are presented as mean ± SEM. iLTP, inhibitory long-term potentiation; INs, interneurons; IPSC; inhibitory postsynaptic current; PV, parvalbumin; SOM, somatostatin.

It has recently been reported that expression of iLTP in cortical pyramidal neurons is input-dependent [44]. Specifically, somatostatin (SOM)-, but not parvalbumin (PV)-, inputs onto prefrontal cortical pyramidal neurons undergo NMDA-induced iLTP. To examine whether iLTP in hippocampal CA1 pyramidal neurons was input-specific, we used an optogenetic approach in which we bilaterally injected adeno-associated virus (AAV) expressing channelrhodopsin-2 (ChR2) fused to mCherry in a Cre recombinase-dependent manner into hippocampal CA1 regions of knock-in mice that expressed Cre under the control of the SOM or PV promoter (SOM-IRES-Cre or PV-IRES-Cre mice) (Fig 4A). Fluorescent images showed that ChR2 expression was highest in stratum lacunosum-moleculare (SLM) layers in SOM-IRES-Cre mice (Fig 4B). Conversely, ChR2 expression was concentrated in the stratum pyramidal (SP) layers in PV-IRES-Cre mice (Fig 4C). These expression profiles confirmed that PV- and SOM- interneurons (INs) respectively target perisomatic and distal dendritic regions of CA1 pyramidal cells [45,46]. We then recorded interneuron subtype-specific inhibitory currents onto CA1 pyramidal neurons by activating ChR2 with 470 nm blue light (Fig 4B and 4C). Surprisingly, we found that inhibitory currents mediated by PV-INs (PV-IPSCs), but not SOM-INs (SOM-IPSCs), exhibited potentiation 30 min post-NMDA application (Fig 4D–4I). Consistently, NMDA application did not cause potentiation of IPSCs evoked by a stimulating electrode placed in SLM (S4A–S4D Fig), but did induce potentiation of IPSCs evoked by stimulation at the perisomatic region (Fig 2E–2H). These findings indicate that inhibitory synapses innervated by PV-INs, but not SOM-INs, contribute to iLTP in hippocampal CA1 pyramidal neurons during sleep and wake states. Additionally, there were no changes of paired-pulse ratio (PPR) of PV-IPSCs before and after NMDA application (S4E and S4F Fig), suggesting that NMDA-induced potentiation of PV-IPSCs is not due to increased probability of presynaptic GABA release at PV-synapses.

The data presented so far showed that PV-iLTP had a higher magnitude in wake mice than sleep mice (Fig 4G–4I). In addition, wake-specific potentiation of mIPSC amplitude was blocked by the α5-GABA_A_R inverse agonist (Fig 3A–3C). Was the higher magnitude of PV-iLTP in wake mice mediated by synaptic recruitment of α5-GABA_A_Rs? To this end, we combined the optogenetic approach with pharmacological assays to determine whether α5-GABA_A_Rs were incorporated into PV-synapses during iLTP in wake mice. Specifically, we perfused α5-GABA_A_R inverse agonist L655,708 20 min post-NMDA application and found that PV-IPSCs in wake mice were decreased to the similar level as that in sleep mice, whereas PV-IPSCs in sleep mice were not affected (Fig 4J–4L). These data indicate that wake-specific enhancement of PV-iLTP is due to recruitment of α5-GABA_A_Rs into PV-synapses. For this experiment, we also set up a group of sleep-deprived mice to distinguish the circadian effects and the sleep/wake effects. These sleep-deprived mice were sacrificed for electrophysiological recordings at the same time as the sleep mice. We found that sleep-deprived mice had increased levels of PV-iLTP compared to sleep mice, and importantly, L655,708 inhibited the additional potentiation, similar to the data collected in wake mice (Fig 4J–4L). These results indicate that sleep-/wake-dependent difference in inhibitory synaptic plasticity is not due to time of the day.

## Discussion

In this study, we have combined behavioral, biochemical, electrophysiological, and optogenetic techniques to investigate the effects of sleep on basal GABAergic transmission and more importantly, inhibitory synaptic plasticity in hippocampal CA1 neurons. We have found that sleep exerts a powerful impact on inhibitory synaptic transmission and plasticity. Specifically, basal inhibitory transmission is lower, but iLTP is significantly higher, in wake mice. Our data have also identified the molecular mechanisms underlying the differential inhibitory synaptic plasticity across sleep and wake and revealed the input-specificity of iLTP in hippocampal neurons.

An influential hypothesis regarding the function of sleep in learning and memory is the synaptic homeostasis hypothesis, which proposes that sleep weakens excitatory synapses and wake strengthens them [3,8]. Indeed, a number of studies have shown molecular, morphological, and electrophysiological changes indicative of synaptic weakening associated with sleep at excitatory synapses [9–11,14,15]. Other studies have also found that excitatory synaptic strength is potentiated [16] or unaffected by sleep [17]. Compared to extensive studies on excitatory synapses [5,6], much less is known about the regulation of inhibitory transmission by the sleep and wake cycle. An early study has shown that evoked IPSC and mIPSC amplitude were higher in cortical pyramidal neurons when measured after short periods of sleep (approximately 20 min) compared to wake [23]. Recently, it has been reported that mIPSC frequency in hippocampal CA1 pyramidal neurons was higher during the light phase (i.e., sleep-dense period) than during the dark phase (i.e., wake-dense period) [25]. However, it is worth noting that the changes induced by day/night cycles do not necessarily reflect sleep-dependent changes. To differentiate the effects of sleep and circadian cycles, we have classified sleep/wake by a non-invasive behavioral tracking system [47] and then measured the changes in inhibitory synaptic strength induced by 4 h natural sleep or wake. We found that both the amplitude and frequency of mIPSCs were higher in hippocampal CA1 pyramidal neurons in sleep than in wake mice, similar to the previous work [25], and consistent with a previous study [23]. Interestingly, we have observed a decrease in tonic inhibition in sleep compared to wake, which is in agreement with our recent work [24]. Currently, the functional consequences of the distinct regulation of phasic and tonic inhibition across sleep and wake remain unclear. It is possible that sleep-associated increase of phasic inhibition contributes to the generation of coherent rhythms of network activity [22,48], which may facilitate memory consolidation during sleep [49–51]. On the other hand, reduced tonic inhibition in sleep may compensate for the changes of phasic inhibition to maintain overall inhibitory tone to preserve the network stability.

Pyramidal neurons in the brain receive functionally distinct GABAergic inputs from different types of interneurons, and many of these GABAergic afferents make domain specific, precise synaptic contacts on pyramidal neurons to provide fine-tuning and dynamic control of pyramidal neuron activity [45,46]. Interestingly, it has been shown that many of these inhibitory synapses can undergo plastic changes in response to different activity patterns [32–34]. However, it remained unknown whether sleep and wake states could modulate inhibitory synapse plasticity in an input-specific manner. Our data now demonstrate that GABAergic synapses in hippocampal CA1 pyramidal neurons from PV- but not SOM-INs undergo NMDA-induced iLTP. Intriguingly, iLTP at PV-synapses shows sleep-dependent modulation. Specifically, PV-iLTP in hippocampal CA1 neurons is higher in wake mice than in sleep mice, indicating that the mechanism underlying PV-iLTP is subject to modulation by sleep and wake. Indeed, NMDA-induced synaptic recruitment of α5-GABA_A_Rs only takes place at PV-synapses in wake, but not sleep mice. Thus, wake enables synaptic trafficking of α5-GABA_A_Rs in response to NMDAR activity in hippocampal neurons. Currently, the molecular pathways that are specifically activated in wake driving synaptic delivery of α5-GABA_A_Rs remain unclear. It has been reported that sleep-dependent regulation of basal synaptic inhibition in cortical neurons depends on activity of voltage-gated calcium channels [23]. It has also been shown that synaptic trafficking of α5-GABA_A_Rs is regulated by a radixin-dependent pathway [42,52]. NMDA-iLTP also depends on the activity of matrix metalloproteinase 3 (MMP3) [38]. In addition, α5-GABA_A_Rs exocytosis and NMDA-iLTP require Shisa7 phosphorylation at S405 [24,53]. Thus, it will be important in the future to examine the roles of voltage-gated calcium channels, radixin, MMP3, and Shisa7 in NMDA-iLTP in hippocampal neurons in wake.

It has been reported that in cortical pyramidal neurons, brief NMDA application selectively drives iLTP at SOM-synapses, while inputs from PV-INs or VIP-INs are unaffected [44]. It has also been shown that theta burst stimulation in the input pathway coupled with postsynaptic depolarization induces T-type calcium channel dependent, but NMDAR-independent, iLTD and iLTP at inhibitory synapses in hippocampal CA1 neurons innervated by PV-INs and SOM-INs, respectively [54]. Additionally, a recent study has demonstrated that induction of excitatory LTP drives α5-GABA_A_Rs into perisynaptic membranes of SOM-synapses in hippocampal CA1 neurons [42]. These data suggest that different types of neurons may express inhibitory synaptic plasticity in an input-specific manner and different induction protocols may engage distinct molecular pathways to trigger synaptic depression, potentiation, or α5-GABA_A_R trafficking at inhibitory synapses. Similar to our data in the present study, NMDA application can induce iLTP of PV-IPSCs in hippocampal cultures [37]. As PV-INs have been shown to play critical roles in learning and memory [55–57], our data that PV-iLTP is regulated by sleep and wake states may help delineate the potential role of PV-INs in sleep or wake-dependent memory processing.

Synaptic plasticity plays a critical role in learning and memory. While the involvement of plasticity at excitatory synapses is well documented, the role of inhibitory synaptic plasticity in learning and memory is much less studied. A recent study has shown that MMP3 is required for iLTP, and genetic deletion of MMP3 enhances hippocampus-dependent spatial learning [38]. Interestingly, our work has identified an important role of α5-GABA_A_R trafficking to synapses in iLTP in wake mice. Accumulating evidence has shown that α5-GABA_A_Rs play pivotal roles in modulating hippocampus-dependent learning and memory [18,58]. Indeed, pharmacological or genetic inhibition of α5-GABA_A_Rs improve hippocampus-dependent learning and memory [59–62], likely through the modulation of synaptic plasticity at excitatory synapses. Specifically, suppression of α5-GABA_A_Rs may reduce the threshold for the induction of excitatory LTP [63] and increase the accumulation of excitatory LTP in hippocampal CA1 pyramidal neurons, facilitating learning [42]. Thus, it is conceivable that wake-specific trafficking of α5-GABA_A_Rs to inhibitory synapses might be an important mechanism to fine tune excitatory synaptic plasticity, which in turn regulates learning and memory during the wake state. Future work examining the role of input-specific inhibitory plasticity in sleep and wake in learning and memory will be invaluable for understanding how sleep regulates memory.

In summary, we have shown a critical role of sleep in inhibitory synaptic transmission and plasticity. We have also revealed sleep-/wake-dependent, input-specific inhibitory plasticity in hippocampal neurons and identified molecular mechanisms underlying the differential inhibitory synaptic plasticity across sleep and wake. These data pave the way for future studies aimed at understanding the function of inhibitory synaptic plasticity in memory across sleep and wake.

## Materials and methods

### Ethics statements

All animal handling was performed in accordance with animal protocols approved by the Institutional Animal Care and Use Committee (IACUC) at NIH/NINDS (protocol numbers: 1339 and 1342).

### Animals

All mice were housed and bred in a conventional vivarium with ad libitum access to food and water under a 12-h circadian cycle (ZT0 - “light on” at 0600, ZT12 - “lights off” at 1800). The wild-type mice (C57BL/6, Strain#: 027) used for experiments were obtained from CHARLES RIVER LABORATORIES. The PV-IRES-CRE (Strain#: 008069) and SOM-IRES-CRE (Strain#: 013044) mice were purchased from the Jackson Laboratory and bred for optogenetic experiments at the home institution. Male mice at 8 to 12 weeks old were used for sleep monitoring, biochemical, and electrophysiological experiments.

### Electrophysiology

Mice were deeply anesthetized with isoflurane (within 1 min) and sacrificed for slicing. Transverse hippocampal slices (300 μM thickness) were obtained in chilled high sucrose cutting solution that contained (in mM): 2.5 KCl, 0.5 CaCl_2_, 7 MgCl_2_, 1.25 NaH_2_PO_4_, 25 NaHCO_3_, 7 glucose, 210 sucrose, and 1.3 ascorbic acid. The slices were recovered in artificial cerebrospinal fluid (ACSF) containing (in mM): 119 NaCl, 2.5 KCl, 26.2 NaHCO_3_, 1 NaH_2_PO_4_, 11 glucose, 2.5 CaCl_2_, and 1.3 MgSO_4_ (pH 7.3; osmolality 300 to 310 mOsm) at 32°C for 30 min and then were maintained at room temperature prior to recording. The internal solution contained (in mM): 70 CsMeSO4, 70 CsCl, 8 NaCl, 10 HEPES, 0.3 NaGTP, 4 Mg-ATP, and 0.3 EGTA (pH 7.3; osmolality 285 to 290 mOsm). mIPSCs were recorded at a holding potential of −70 mV in the presence of 0.5 μM TTX and 20 μM DNQX. For experiments involving local electrical stimulation, a glass theta stimulating electrode was filled with ACSF and was placed in distal or perisomatic areas of hippocampal CA1 regions to evoke IPSCs (eIPSCs). eIPSCs were recorded at a holding potential of −70 mV in the presence of DNQX (20 μM). For both mIPSCs and eIPSCs, iLTP was induced by transient NMDA (3 min, 20 μM) bath application after stable baseline recordings. The NMDA was then washed out, and mIPSCs or eIPSCs were recorded for another 30 min. During the application of NMDA, the cells were voltage-clamped, as described before [44]. The data were binned into 2-min time bins and then averaged to mean amplitude. The extent of iLTP was defined as the ratio of the mean amplitude recorded 30 min after NMDA application to the amplitude recorded before NMDA application. In some recordings as indicated, L-655,708 (100 nM, Sigma-Aldrich) was added to the ACSF via perfusion. To record tonic currents, cells were patch-clamped under the voltage-clamp mode at a holding potential of −70 mV in the presence of DNQX (20 μM). After stable baseline recordings, the GABA_A_R competitive antagonist bicuculline (20 μM, Abcam) was bath applied. The difference in baseline holding currents before and during bicuculline application was calculated to be the tonic currents. Series resistance was monitored and not compensated, and cells in which series resistance was more than 25 MΩ or varied by 25% during a recording session were discarded. Data were collected with a Multiclamp 700B amplifier (Axon Instruments), filtered at 2 kHz, and digitized at 10 kHz. All recordings were conducted at room temperature in a submersion-type recording chamber. Offline analysis was carried out in Igor Pro (Wavemetrics) as described previously [64].

### Synaptic stimulation

To express ChR2 in PV- or SOM-positive interneurons, PV-IRES-Cre mice or SOM-IRES-Cre mice were injected bilaterally into the hippocampal CA1 region (PV-IRES-Cre, AP: −1.45 mm, ML: ±1.26 mm, DV: −1.45 mm; SOM-IRES-Cre, AP: −1.40 mm, ML: ±1.25 mm, DV: −1.3 mm) with recombinant AAV driving Cre-dependent expression of ChR2-mCherry (AAV5-EF1a-DIO-hChR2(H134R)-mCherry, Addgene, Cat#: 20297). Mice were anesthetized under inhaled 1.5% isoflurane for the surgical procedure and allowed to fully recover from anesthesia on a warmed pad. Mice were sacrificed 16 to 25 days post injection for slice preparation as described above. IPSCs were evoked using pulses of blue light (3 ms, 0.1 to 3 mW, 20-s interstimulus interval), through a 40× objective. NMDA-induced iLTP of IPSCs was performed as described above. PPRs were examined using optogenetically evoked IPSCs at 50-ms intervals.

### Piezoelectric sleep recording

Sleep–wake activity was recorded using a piezoelectric monitoring system (Signal Solutions) as described with minor modifications [47,65]. It has been demonstrated that this sensitive system estimated total sleep time with more than 90% accuracy compared to EEG, although it cannot distinguish rapid eye movement (REM) sleep from non-rapid eye movement (NREM) [47]. Thus, our work did not intend to study the effect of different sleep stages on inhibitory transmission, which will need to combine EEG recordings with in vitro slice physiology. Instead, our work aimed to investigate how inhibitory transmission is altered during sleep or wake in general regardless of its stages. Prior to piezoelectric recording, 8 to 12 weeks old male mice were singly housed and habituated to the recording cage with free access to food and water for 2 days under a 12-h circadian cycle. During piezoelectric recording, mice were left undisturbed and the piezoelectric signals in 2-s epochs were automatically analyzed by a linear discriminant classifier algorithm and classified as sleep or wake. Total sleep percentages and hourly sleep percentages were calculated using SleepStats Data Explorer (Signal Solutions). Based on the sleep pattern we recorded (Fig 1) and the criteria used in the previous studies [11,24], we defined sleep/wake mice as follows: Sleep mice were asleep at least 65% of the previous 4 h (at least 60% per hour). Wake mice were awake at least 75% of the previous 4 h (at least 70% per hour). Mice were selected for electrophysiology experiments only if they met the criteria. Each individual mouse has their own sleep–wake pattern and they may be collected at different times of day based on our sleep/wake definition (Fig 1C and 1D). Sleep mice were sacrificed during the light phase (ZT6-9) and wake mice were sacrificed during the dark phase (ZT16-19). For some experiments, mice were sleep-deprived by gently handling as described previously [24] for 4 h during the light phase, when they would normally have slept. These sleep-deprived mice were then sacrificed for electrophysiological recordings at the same time with the sleep mice.

### Synaptosome preparation

Synaptosomes were purified according to previously described methods [66]. Briefly, the hippocampi were homogenized in Sucrose/EDTA/DTT buffer (0.32 M Sucrose, 1 mM EDTA, 0.25 mM DTT, and 5 mM Tris (pH 7.4)). The homogenate was first centrifuged at 1,000 g for 10 min at 4°C to remove nuclei and debris; the supernatant was then gently layered on a discontinuous Percoll gradient (3%, 10%, 15%, and 23% v/v in Sucrose/EDTA/DTT buffer) and then centrifuged at 31,000 g for 6 min at 4°C. The synaptosomal fractions were collected from the layer between 15% and 23% and washed by centrifugation at 20,000 g for 30 min at 4°C. After wash, the synaptosomal samples were collected for western blot analysis.

### Surface biotinylation assay

Surface biotinylation was performed as described before [67]. Briefly, hippocampal slices were incubated with ACSF containing 1 mg/ml sulfo-NHS-SS biotin (Cat# 21331, Thermo Fisher Scientific) for 30 min at 4°C. Slices were then washed with ACSF and unreacted biotin was quenched by washing slices 3 times with ACSF containing 100 mM Glycine (pH 7.4) and collected in lysis buffer (25 mM Tris (pH 7.5), 1% Triton X-100, 150 mM NaCl, 5% glycerol, 1 mM EDTA, and protease inhibitor cocktail). Lysates were clarified by centrifugation at 12,000 g for 15 min at 4°C, and the protein concentrations measured using BCA Protein Assay Kit (Thermo Fisher Scientific). The biotinylated proteins were precipitated with streptavidin agarose resin (Cat# 20353, Thermo Fisher Scientific). Total proteins and surface proteins were analyzed with western blot.

### Western blot

The hippocampi were dissected and homogenized in lysis buffer (25 mM Tris (pH 7.5), 1% Triton X-100, 150 mM NaCl, 5% glycerol, 1 mM EDTA, and protease inhibitor cocktail). Lysates were clarified by centrifugation at 12,000 g for 15 min at 4°C, and the protein concentrations measured using BCA Protein Assay Kit (Thermo Fisher Scientific). Equal amounts of protein (approximately 15 μg) were loaded in each lane of the individual gels. The protein was separated by SDS-PAGE gels (BioRad) and then transferred onto PVDF membranes. The membranes were blocked with 5% milk for 1 h at room temperature (23°C), incubated with primary antibodies at 4°C overnight, washed and incubated with HRP-conjugated secondary antibodies (HRP-conjugated Goat Anti-Mouse IgG, Cat# 111-035-144 or HRP-conjugated Goat Anti-Rabbit IgG, Cat# 115-035-062, Jackson ImmunoResearch Laboratories) for 1 h at room temperature (23°C). Protein was detected with the standard enhanced chemiluminescence (ECL) method and documented by a gel imaging system (Li-COR Odyssey). The blot images were analyzed by ImageJ software (NIH).

The following primary antibodies were used: Rabbit Polyclonal Anti-GABA(A) α1 Receptor (α1) (Cat# 06–868, Millipore), Rabbit Polyclonal Anti-GABA(A) α2 Receptor (α2) (Cat# 224103, Synaptic Systems), Rabbit Polyclonal Anti-GABA(A) β3 Receptor (β3) (Cat# SAB2100880, Sigma-Aldrich), Rabbit Polyclonal Anti-GABA(A) α4 Receptor (α4) (Cat# AGA-008, Alomone Labs), Rabbit Polyclonal Anti-GABA(A) α5 Receptor (α5) (Cat# 224503, Synaptic Systems), Mouse monoclonal anti-Gephyrin (Cat# 147021, Synaptic Systems), Mouse monoclonal α-tubulin (Cat# T8203, Sigma-Aldrich), Mouse monoclonal Anti-Glutamate Receptor NMDAR1 (GluN1) (Cat# MAB363, Sigma-Aldrich), Rabbit Polyclonal Anti-Glutamate Receptor NMDAR2A (GluN2A) (Cat# M264, Sigma-Aldrich), and Rabbit Polyclonal Anti-Glutamate Receptor NMDAR2B (GluN2B) (Cat# M265, Sigma-Aldrich).

### Statistical analysis

For all biochemical and electrophysiological recordings, at least 3 independent experiments were performed. Statistical analysis was performed in GraphPad Prism 8.0 software. Direct comparisons between 2 groups were made using 2-tailed Student *t* test. Multiple comparisons were performed using 2-way ANOVA with corrections for multiple comparisons test (see figure legends for specifics). The statistical significance was defined as *p < 0.05, **p < 0.01, ***p < 0.001, or ****p < 0.0001, respectively. All data are presented as mean ± SEM.

## Supporting information

S1 Fig GABA_A_Rs expression and α5-GABA_A_R-mediated tonic currents change across sleep and wake states. **Related to Fig 1.** (A, B) Representative immunoblots of GABA_A_Rs extracted from the hippocampus of sleep and wake mice. Total proteins from the homogenates (A) and synaptosome fractions (B) were analyzed by western blotting. (C) Summary graphs showing that there was no change of GABA_A_ receptor subunits in the total homogenates across sleep and wake (*n* = 4 independent experiments, *t* test). (D) Summary graphs showing that wake inhibited synaptic α1/α2-GABA_A_R expression but promoted extrasynaptic α4/α5-GABA_A_R expression in the synaptosomes (*n* = 4 independent experiments, *t* test, α1, *p* = 0.013; α2, *p* = 0.0021; α4, *p* = 0.0004; α5, *p* = 0.0084). (E–G) Representative immunoblots and summary graphs from cell-surface biotinylation assays showing increased α4/α5-GABA_A_R expression in cell surface membrane (*n* = 4 independent experiments, *t* test, α4, *p* = 0.039; α5, *p* = 0.03). (H–J) Wake increased α5-GABA_A_Rs-mediated tonic inhibition in CA1 pyramidal neurons in acute hippocampal slices. L655,708 (100 nM), an inverse agonist of α5-GABA_A_Rs, was applied to block α5-GABA_A_Rs-mediated tonic currents before blocking all GABA_A_Rs with bicuculline during recording. L-655,708-sensitive components, but not L-655,708-insensitive components, of tonic currents were significantly increased in wake state. (*n* = 7–8, *t* test, L-655,708-sensitive tonic currents, *p* = 0.027). The data underlying this figure can be found in S1 Data. *p < 0.05, **p < 0.01, and ***p < 0.001. All data are presented as mean ± SEM.
(TIF)

S2 Fig Sleep-/wake-dependent difference in inhibitory synaptic transmission is independent of time of day. **Related to Fig 1.** (A) Representative mIPSC traces from CA1 neurons in acute hippocampal slices prepared from sleep and sleep-deprived mice (SD). (B, C) mIPSC frequency and amplitude were decreased in hippocampal CA1 pyramidal neurons in sleep-deprived mice compared to sleep mice. (*n* = 9–10, *t* test, Frequency, *p* = 0.0003; Amplitude, *p* = 0.0033). (D) There was no difference of mIPSC decay time constants in sleep and sleep-deprived mice. (*n* = 9–10, *t* test). (E) Tonic inhibition was increased in hippocampal CA1 pyramidal neurons in sleep-deprived mice compared to sleep mice. (*n* = 9–10, *t* test, *p* = 0.0083). The data underlying this figure can be found in S1 Data.**p < 0.01 and ***p < 0.001. All data are presented as mean ± SEM.
(TIF)

S3 Fig iLTP is induced by a postsynaptic mechanism. **Related to Fig 2.** (A) Time course of mIPSC amplitude in hippocampal CA1 pyramidal cells before and after NMDA application. BAPTA, a fast Ca^2+^ chelator was applied through the recording pipette. The data were binned into 2-min time bins. (B) Representative mIPSC traces at indicated time points in (A). (C) There were no changes of eIPSC amplitude before and after NMDA application in sleep and wake mice, when BAPTA was applied through the recording pipette (*n* = 5–6, 2-way ANOVA with Sidak’s multiple comparison test). (D–F) Representative western blots and summary graphs from cell-surface biotinylation assays showing that there were no changes of total or surface GluN1, GluN2A, or GluN2B across sleep and wake (*n* = 4 independent experiments, *t* test). The data underlying this figure can be found in S1 Data. All data are presented as mean ± SEM.
(TIF)

S4 Fig Analysis of IPSCs evoked by electrical stimulation in SLM and PPR of PV-IPSCs before and after NMDA application. **Related to Fig 4.** (A) IPSCs evoked by electrical stimulation (eIPSCs) in SLM. (B) Time course of eIPSC amplitude before and after NMDA application. The data were binned into 2-min time bins. (C) Representative eIPSC traces at indicated time points in (F). (D) There were no changes of eIPSC amplitude before and after NMDA application in sleep and wake mice. (*n* = 5–6, 2-way ANOVA with Sidak’s multiple comparison test). (E) Representative PV-IPSC traces evoked by 2 consecutive pulses of blue light at 50-ms intervals pre-NMDA application (Pre) and 20–40 min post-NMDA application (Post). (F) There were no changes of PPR of PV-IPSCs before and after NMDA application in sleep and wake mice (n = 6, 2-way ANOVA with Sidak’s multiple comparison test). The data underlying this figure can be found in S1 Data.
(TIF)

S1 Data Numerical data underlying Figs 1–4 and S1–S4. Excel spreadsheet containing in separate sheets the numerical data underlying Figs 1–4 and S1–S4, respectively.
(XLSX)

S1 Raw Images Original and uncropped western blots data for S1A, S1B, S1E, and S3D Figs. (ZIP)

## Abbreviations

AAV: adeno-associated virus
ACSF: artificial cerebrospinal fluid
ECL: enhanced chemiluminescence
IACUC: Institutional Animal Care and Use Committee
iLTP: inhibitory long-term potentiation
INs: interneurons
LTP: long-term potentiation
mIPSC: miniature inhibitory postsynaptic current
MMP3: matrix metalloproteinase 3
NREM: non-rapid eye movement
PPR: paired-pulse ratio
PV: parvalbumin
REM: rapid eye movement
SLM: stratum lacunosum-moleculare
SOM: somatostatin
SP: stratum pyramidal
