# Supplementary figures and images for: Sleep and wake cycles dynamically modulate hippocampal inhibitory synaptic plasticity
Source: PLoS Biol. 2022 Nov 1;20(11):e3001812. doi: 10.1371/journal.pbio.3001812 (PMC9624398; doi:10.1371/journal.pbio.3001812)

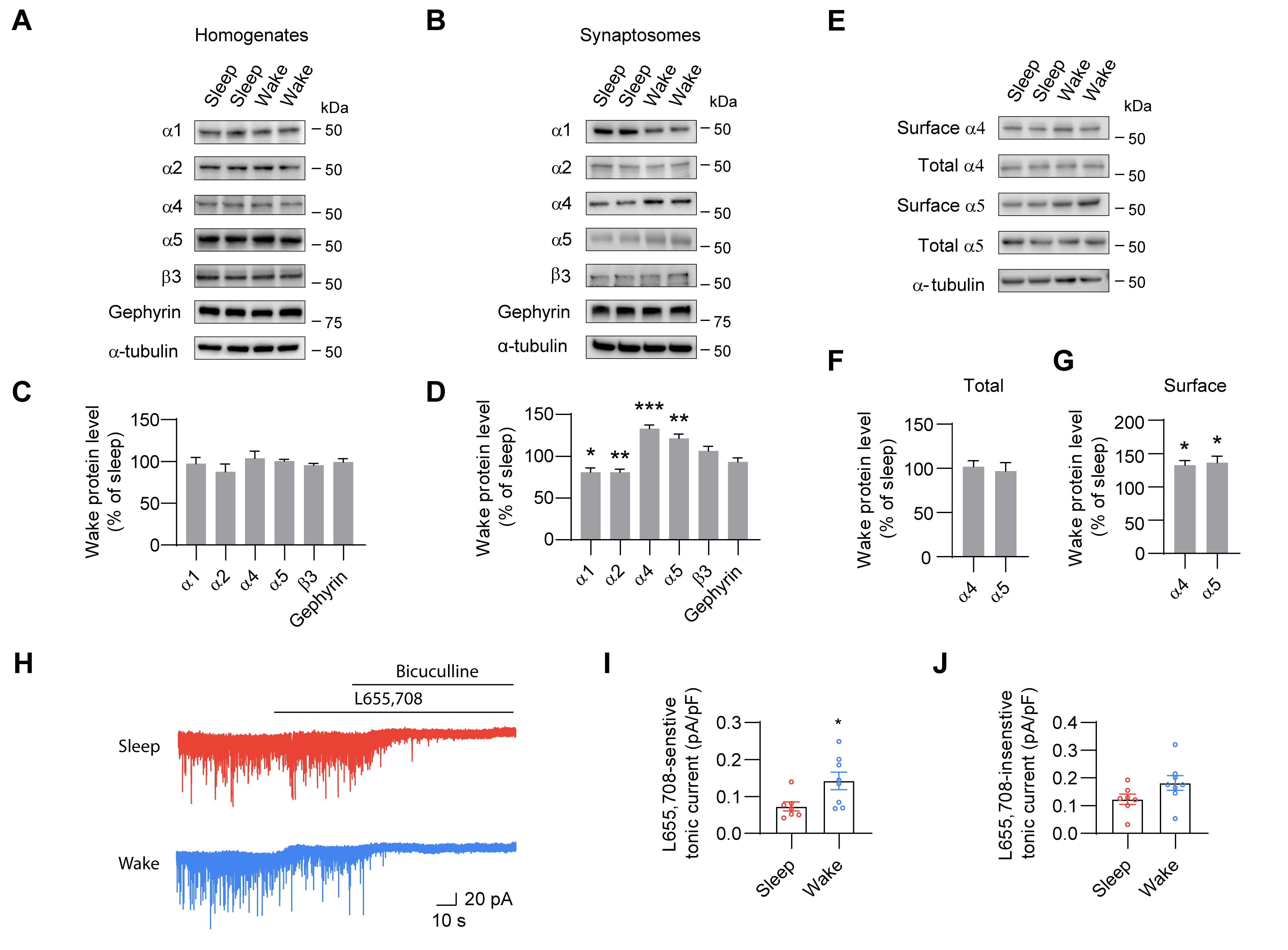

Supplement: S1 Fig — Related to Fig 1. (A, B) Representative immunoblots of GABAARs extracted from the hippocampus of sleep and wake mice. Total proteins from the homogenates (A) and synaptosome fractions (B) were analyzed by western blotting. (C) Summary graphs showing that there was no change of GABAA receptor subunits in the total homogenates across sleep and wake (n = 4 independent experiments, t test). (D) Summary graphs showing that wake inhibited synaptic α1/α2-GABAAR expression but promoted extrasynaptic α4/α5-GABAAR expression in the synaptosomes (n = 4 independent experiments, t test, α1, p = 0.013; α2, p = 0.0021; α4, p = 0.0004; α5, p = 0.0084). (E–G) Representative immunoblots and summary graphs from cell-surface biotinylation assays showing increased α4/α5-GABAAR expression in cell surface membrane (n = 4 independent experiments, t test, α4, p = 0.039; α5, p = 0.03). (H–J) Wake increased α5-GABAARs-mediated tonic inhibition in CA1 pyramidal neurons in acute hippocampal slices. L655,708 (100 nM), an inverse agonist of α5-GABAARs, was applied to block α5-GABAARs-mediated tonic currents before blocking all GABAARs with bicuculline during recording. L-655,708-sensitive components, but not L-655,708-insensitive components, of tonic currents were significantly increased in wake state. (n = 7–8, t test, L-655,708-sensitive tonic currents, p = 0.027). The data underlying this figure can be found in S1 Data. *p < 0.05, **p < 0.01, and ***p < 0.001. All data are presented as mean ± SEM. (TIF) [file pbio.3001812.s001.tif]

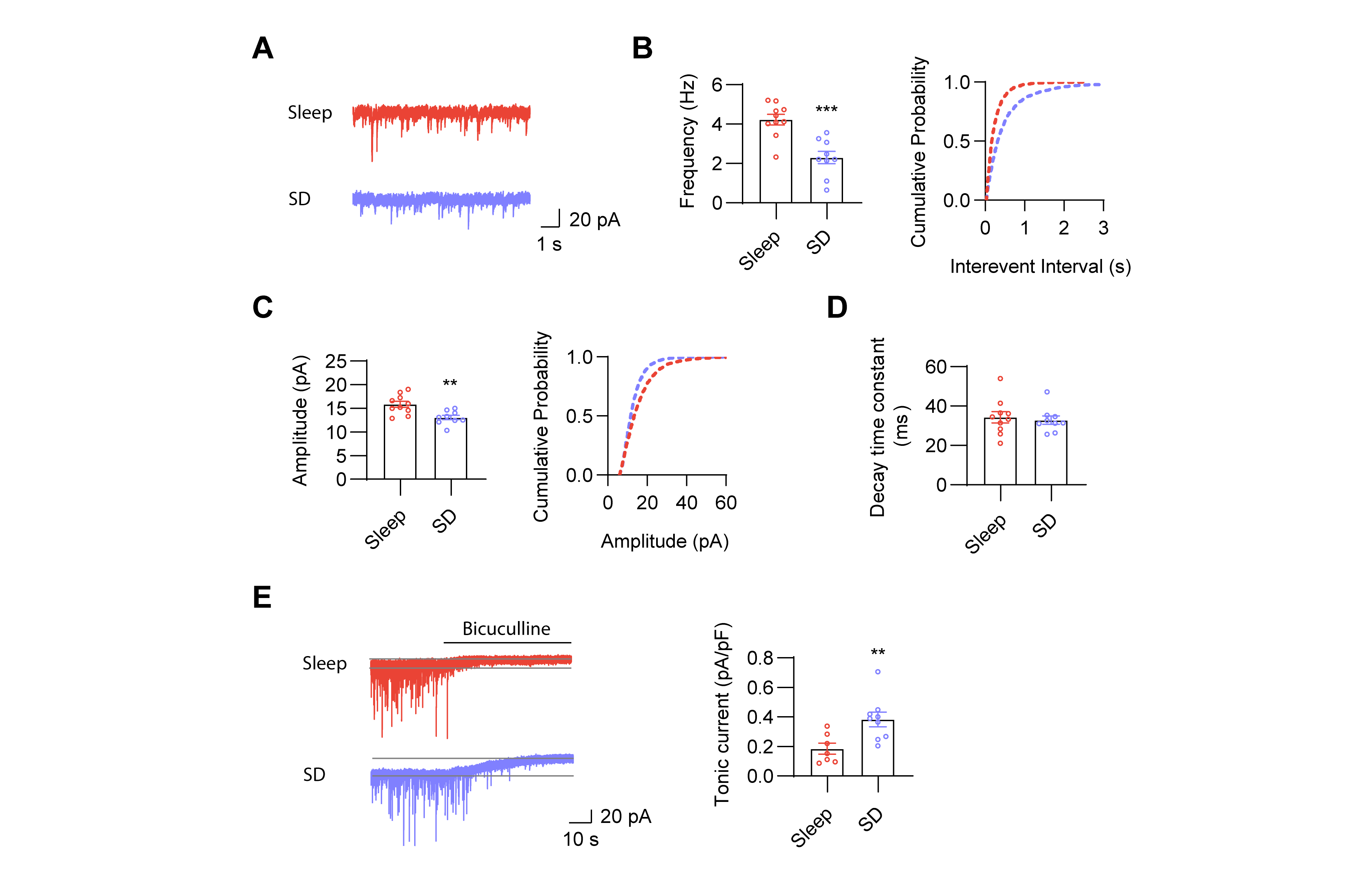

Supplement: S2 Fig — Related to Fig 1. (A) Representative mIPSC traces from CA1 neurons in acute hippocampal slices prepared from sleep and sleep-deprived mice (SD). (B, C) mIPSC frequency and amplitude were decreased in hippocampal CA1 pyramidal neurons in sleep-deprived mice compared to sleep mice. (n = 9–10, t test, Frequency, p = 0.0003; Amplitude, p = 0.0033). (D) There was no difference of mIPSC decay time constants in sleep and sleep-deprived mice. (n = 9–10, t test). (E) Tonic inhibition was increased in hippocampal CA1 pyramidal neurons in sleep-deprived mice compared to sleep mice. (n = 9–10, t test, p = 0.0083). The data underlying this figure can be found in S1 Data.**p < 0.01 and ***p < 0.001. All data are presented as mean ± SEM. (TIF) [file pbio.3001812.s002.tif]

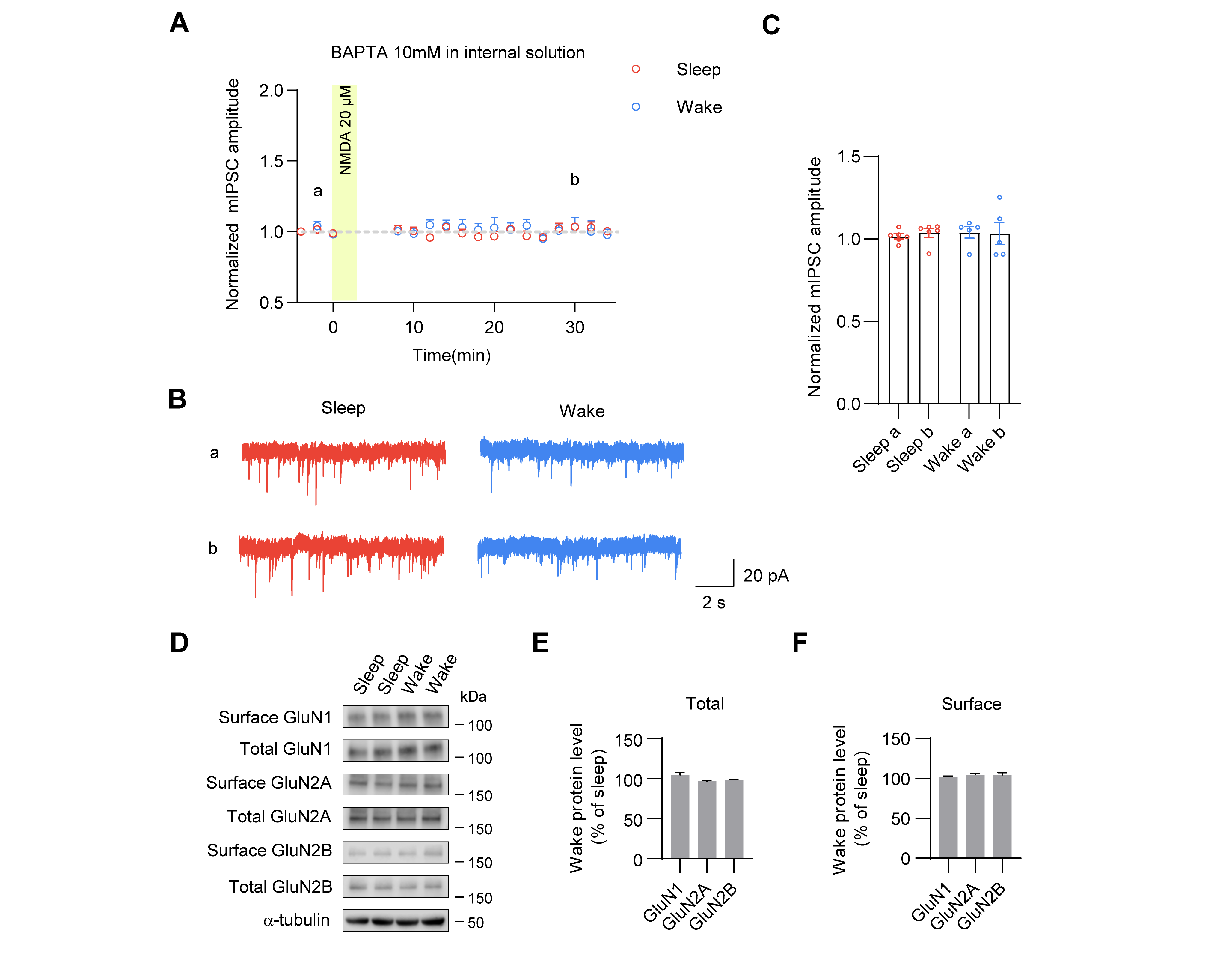

Supplement: S3 Fig — Related to Fig 2. (A) Time course of mIPSC amplitude in hippocampal CA1 pyramidal cells before and after NMDA application. BAPTA, a fast Ca2+ chelator was applied through the recording pipette. The data were binned into 2-min time bins. (B) Representative mIPSC traces at indicated time points in (A). (C) There were no changes of eIPSC amplitude before and after NMDA application in sleep and wake mice, when BAPTA was applied through the recording pipette (n = 5–6, 2-way ANOVA with Sidak’s multiple comparison test). (D–F) Representative western blots and summary graphs from cell-surface biotinylation assays showing that there were no changes of total or surface GluN1, GluN2A, or GluN2B across sleep and wake (n = 4 independent experiments, t test). The data underlying this figure can be found in S1 Data. All data are presented as mean ± SEM. (TIF) [file pbio.3001812.s003.tif]

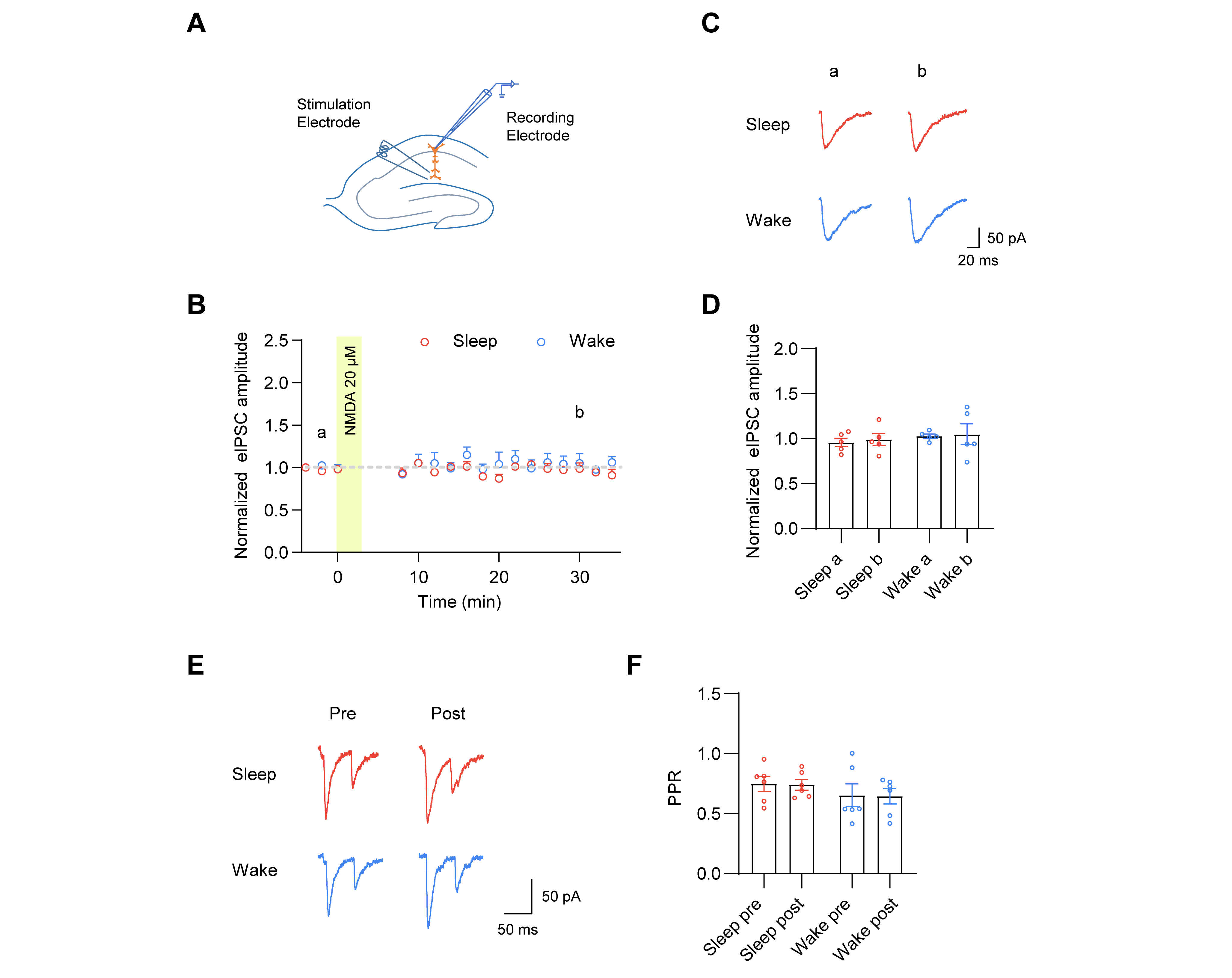

Supplement: S4 Fig — Related to Fig 4. (A) IPSCs evoked by electrical stimulation (eIPSCs) in SLM. (B) Time course of eIPSC amplitude before and after NMDA application. The data were binned into 2-min time bins. (C) Representative eIPSC traces at indicated time points in (F). (D) There were no changes of eIPSC amplitude before and after NMDA application in sleep and wake mice. (n = 5–6, 2-way ANOVA with Sidak’s multiple comparison test). (E) Representative PV-IPSC traces evoked by 2 consecutive pulses of blue light at 50-ms intervals pre-NMDA application (Pre) and 20–40 min post-NMDA application (Post). (F) There were no changes of PPR of PV-IPSCs before and after NMDA application in sleep and wake mice (n = 6, 2-way ANOVA with Sidak’s multiple comparison test). The data underlying this figure can be found in S1 Data. (TIF) [file pbio.3001812.s004.tif]

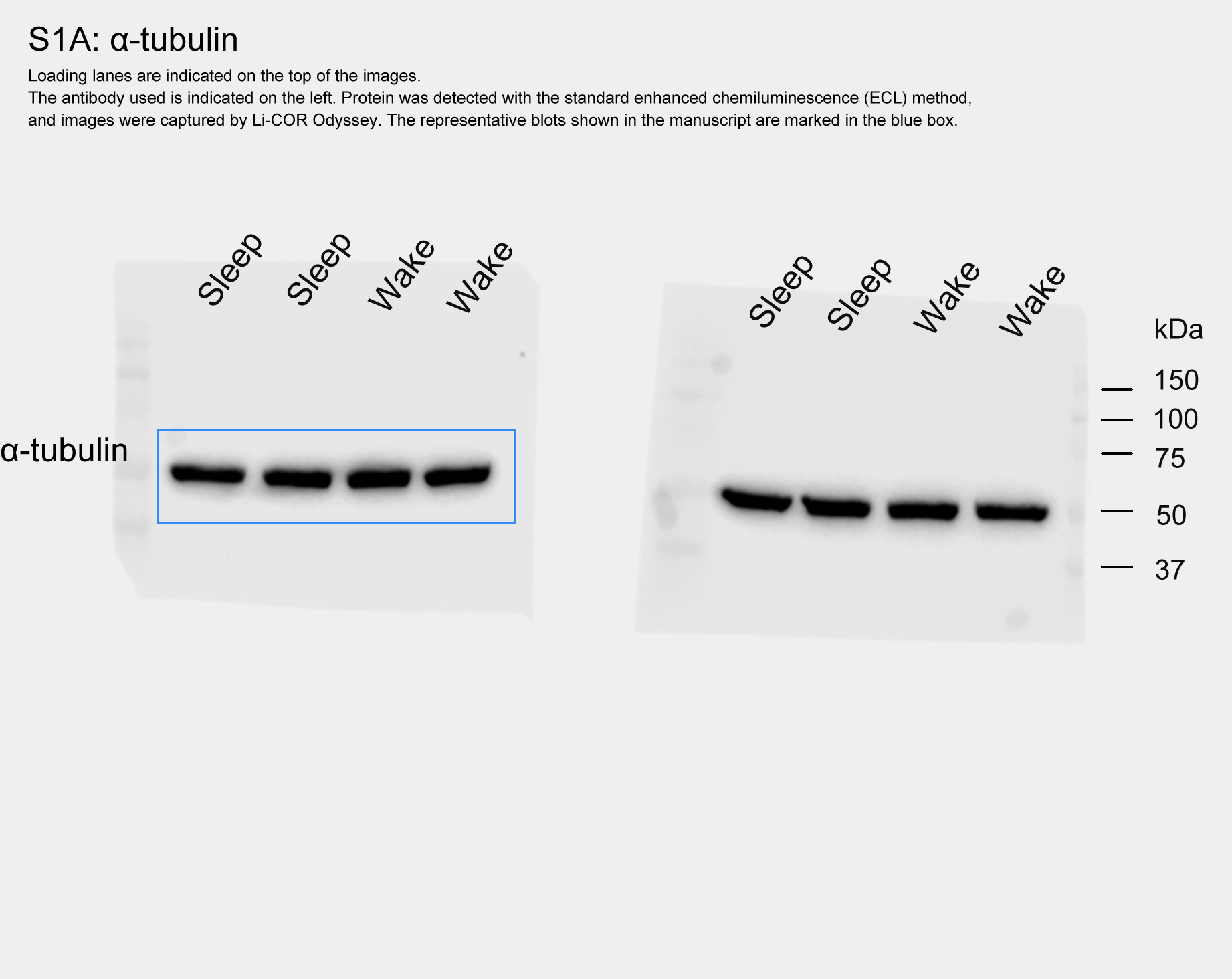

Supplement: S1 Raw Images — (ZIP) [file pbio.3001812.s006.zip › S1_raw_images/S1A/S1A-a-tubulin.tif]

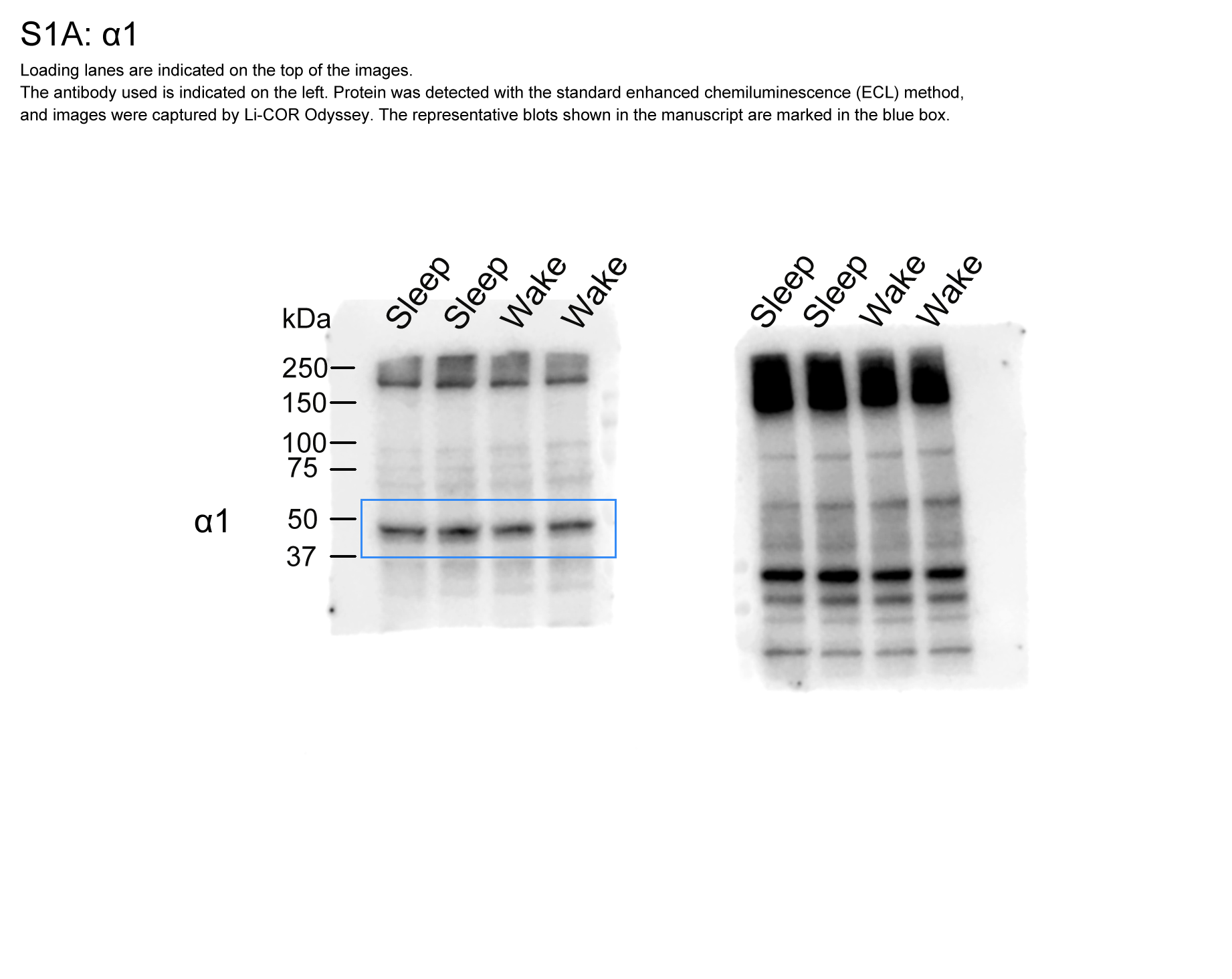

Supplement: S1 Raw Images — (ZIP) [file pbio.3001812.s006.zip › S1_raw_images/S1A/S1A-a1.tif]

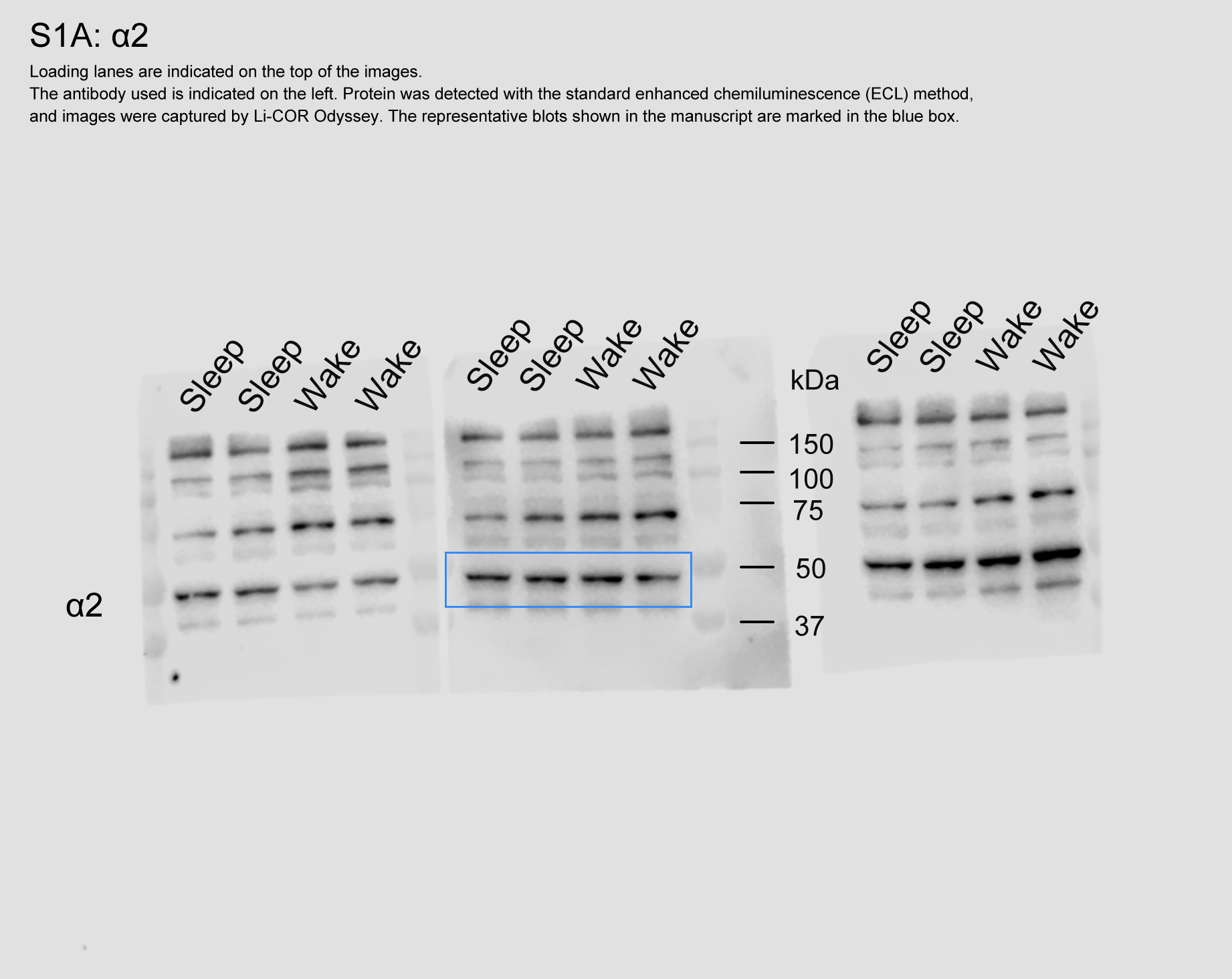

Supplement: S1 Raw Images — (ZIP) [file pbio.3001812.s006.zip › S1_raw_images/S1A/S1A-a2.tif]

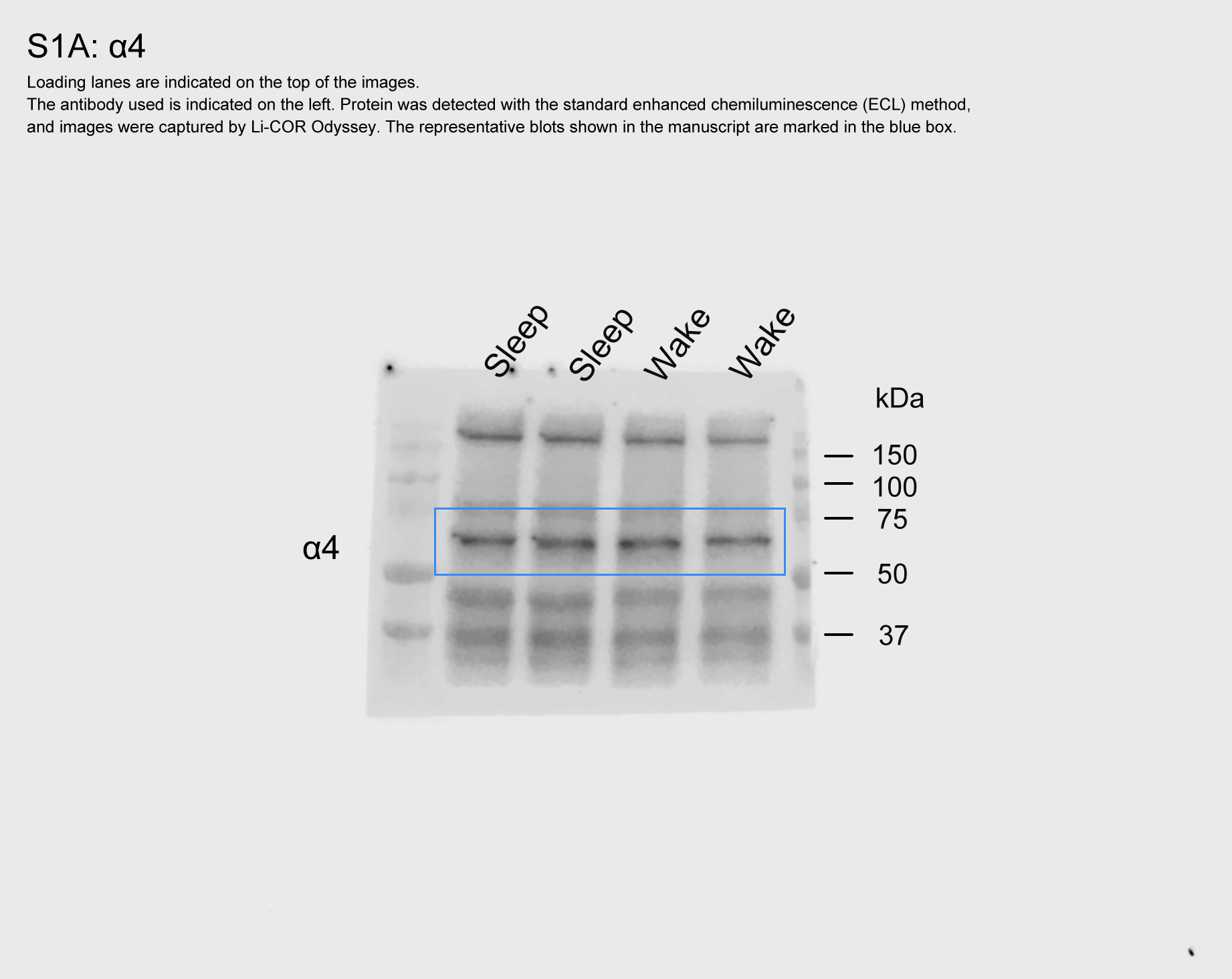

Supplement: S1 Raw Images — (ZIP) [file pbio.3001812.s006.zip › S1_raw_images/S1A/S1A-a4.tif]

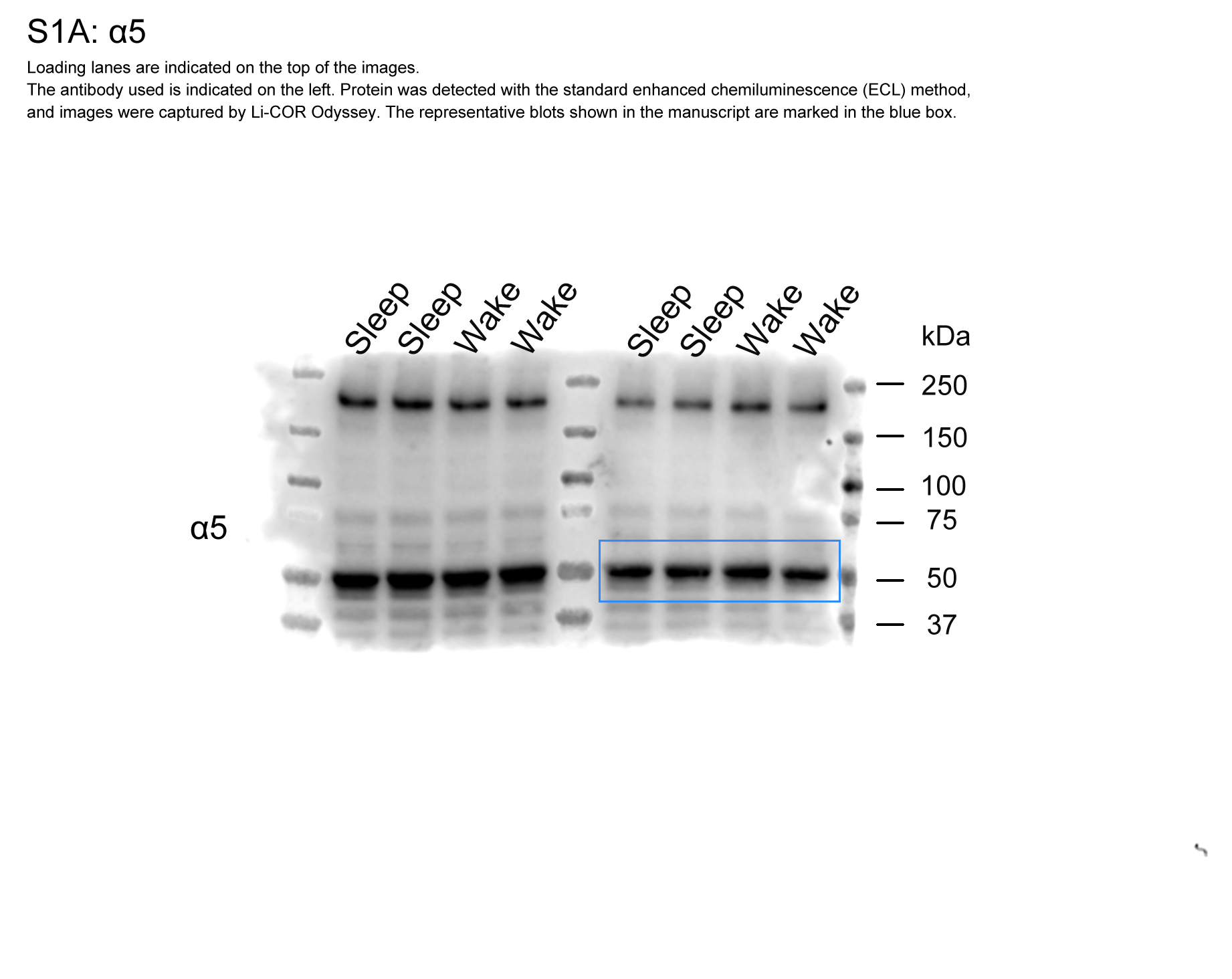

Supplement: S1 Raw Images — (ZIP) [file pbio.3001812.s006.zip › S1_raw_images/S1A/S1A-a5.tif]

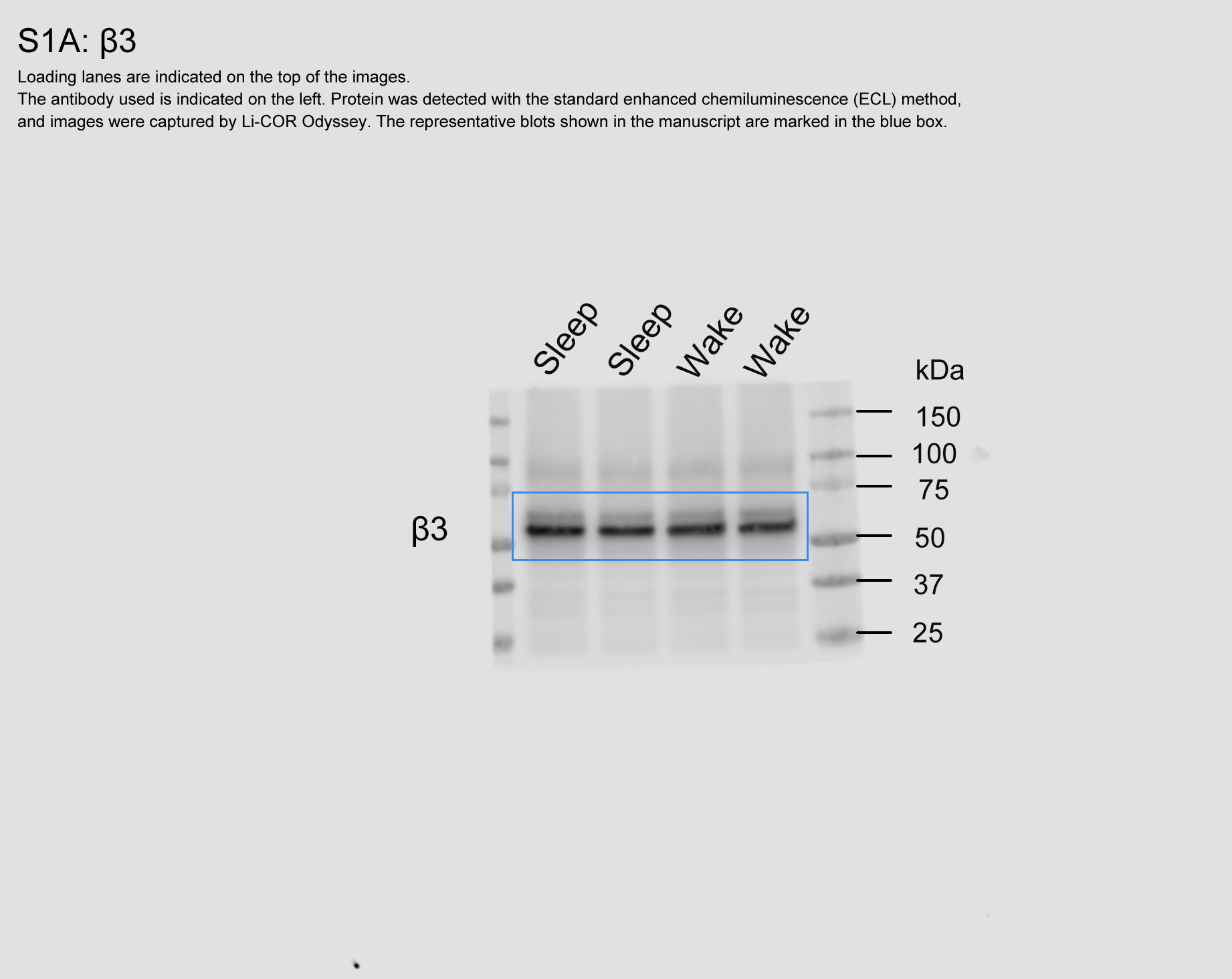

Supplement: S1 Raw Images — (ZIP) [file pbio.3001812.s006.zip › S1_raw_images/S1A/S1A-beta3.tif]

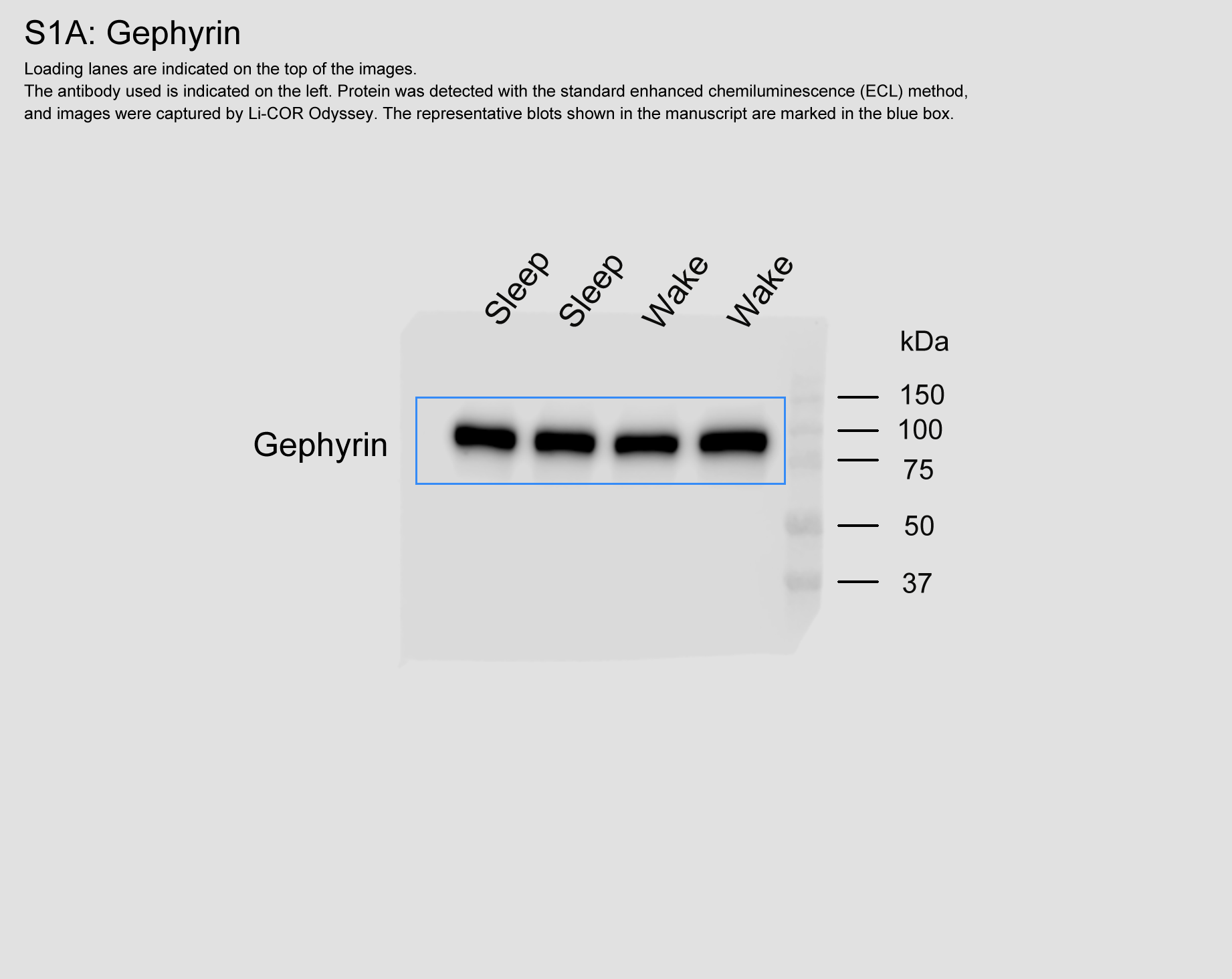

Supplement: S1 Raw Images — (ZIP) [file pbio.3001812.s006.zip › S1_raw_images/S1A/S1A-gephyrin.tif]

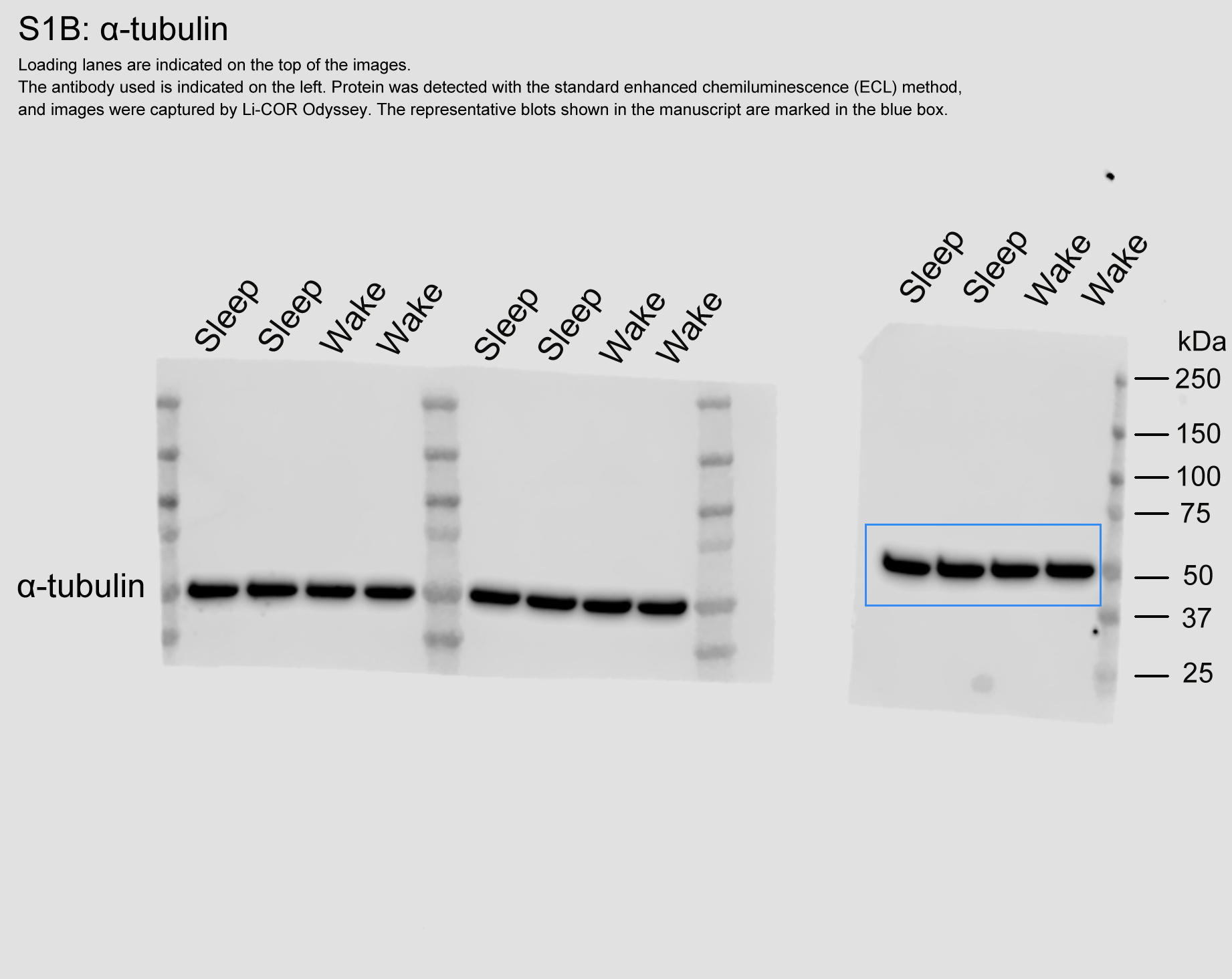

Supplement: S1 Raw Images — (ZIP) [file pbio.3001812.s006.zip › S1_raw_images/S1B/S1B-a-tubulin.tif]

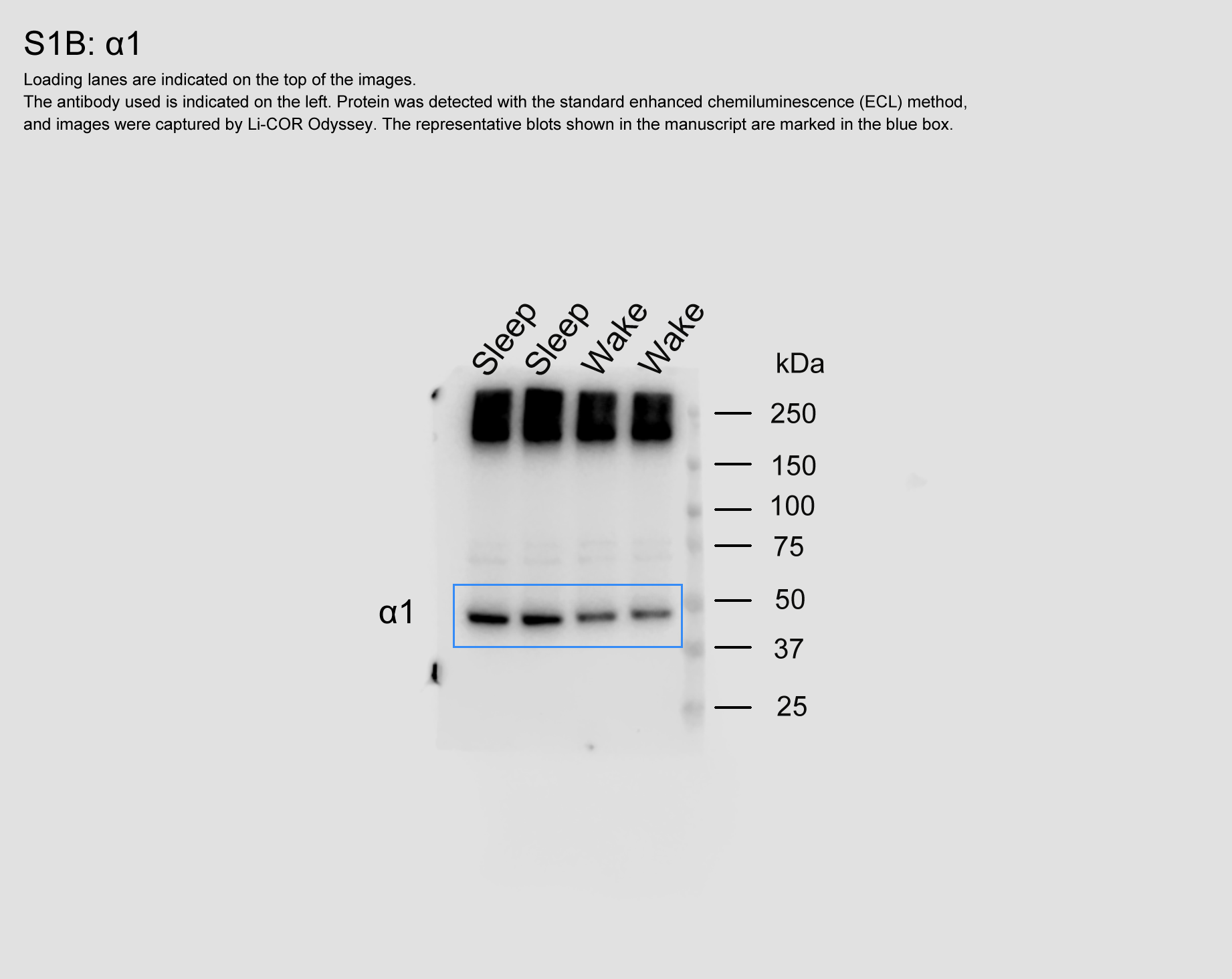

Supplement: S1 Raw Images — (ZIP) [file pbio.3001812.s006.zip › S1_raw_images/S1B/S1B-a1.tif]

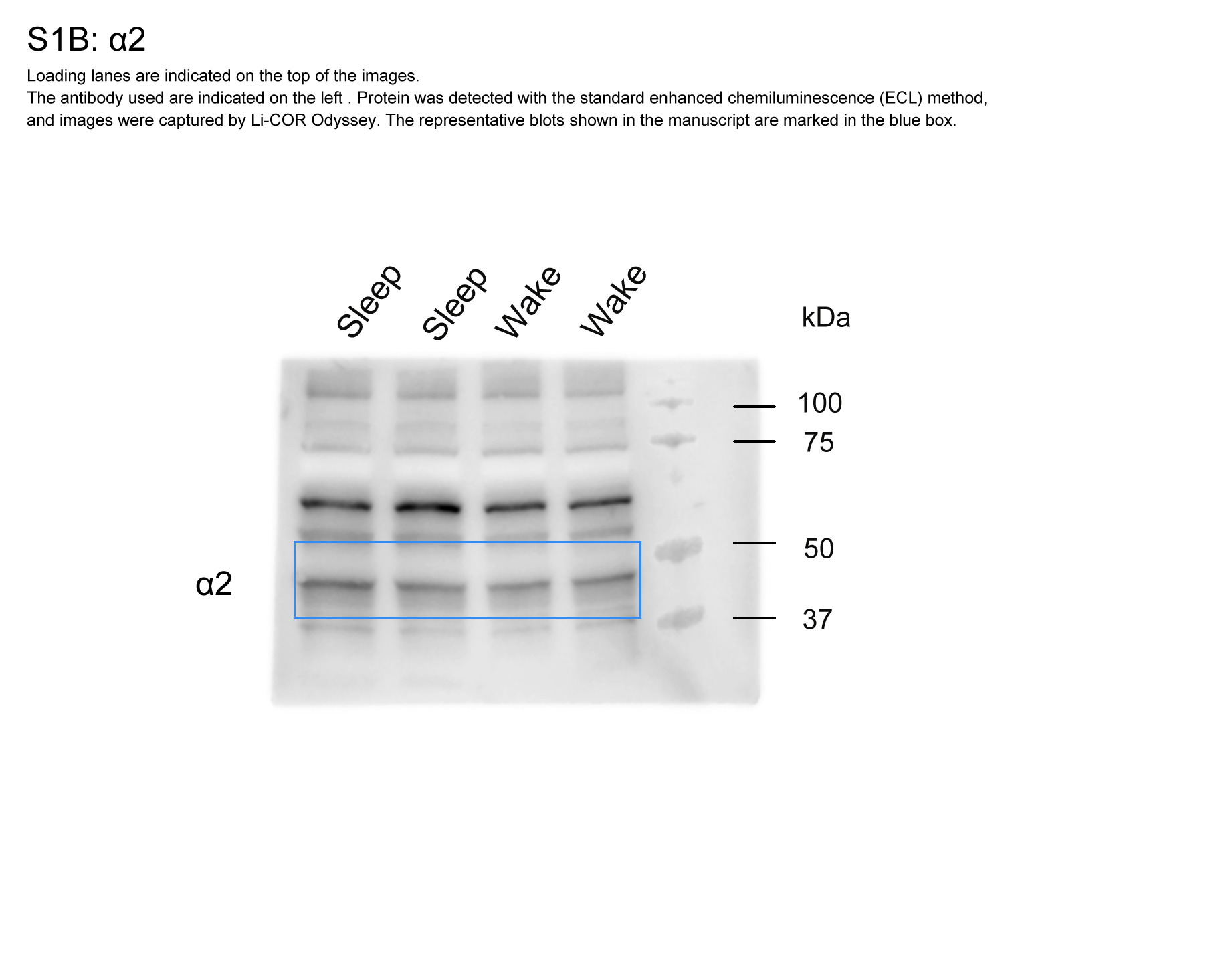

Supplement: S1 Raw Images — (ZIP) [file pbio.3001812.s006.zip › S1_raw_images/S1B/S1B-a2.tif]

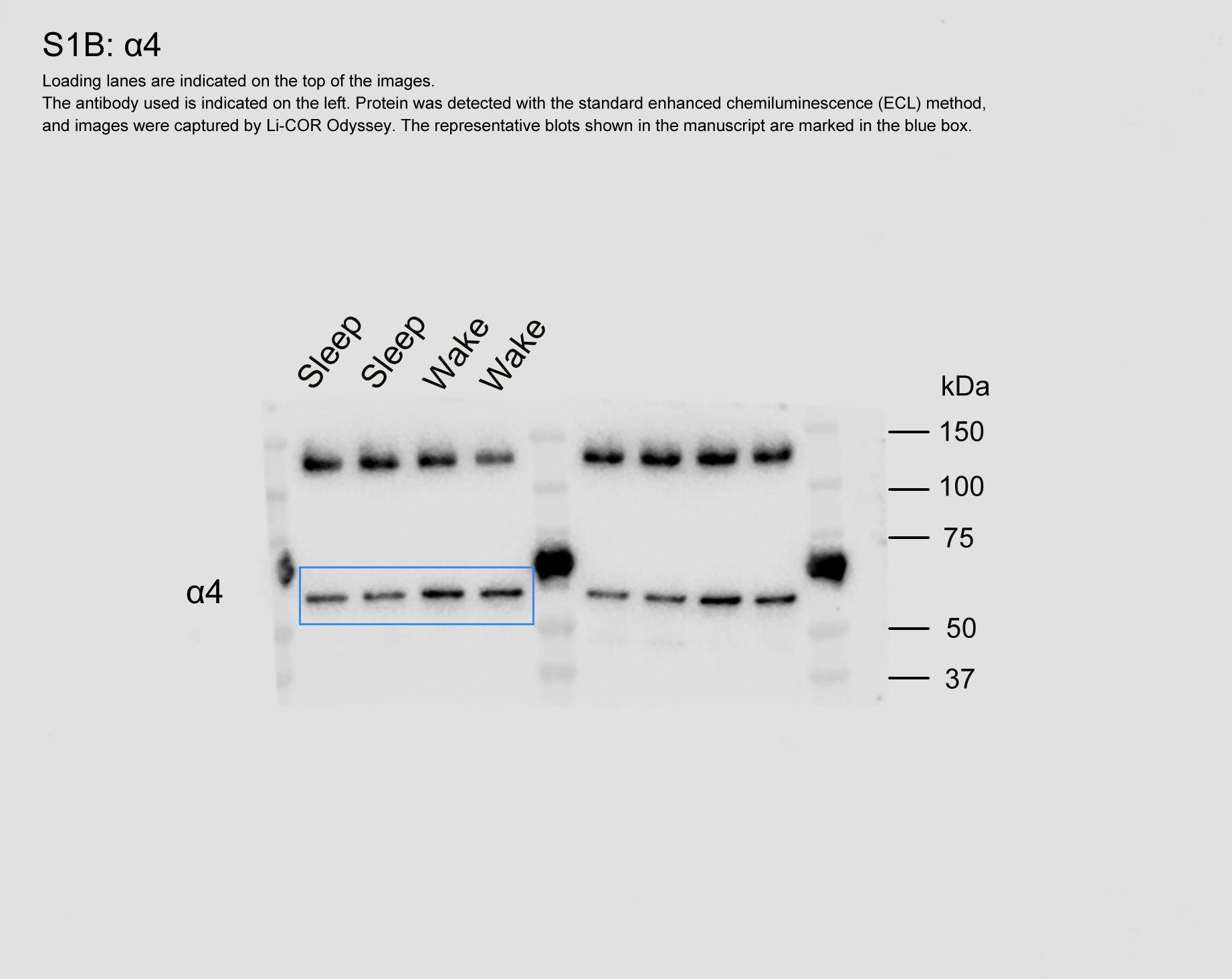

Supplement: S1 Raw Images — (ZIP) [file pbio.3001812.s006.zip › S1_raw_images/S1B/S1B-a4.tif]

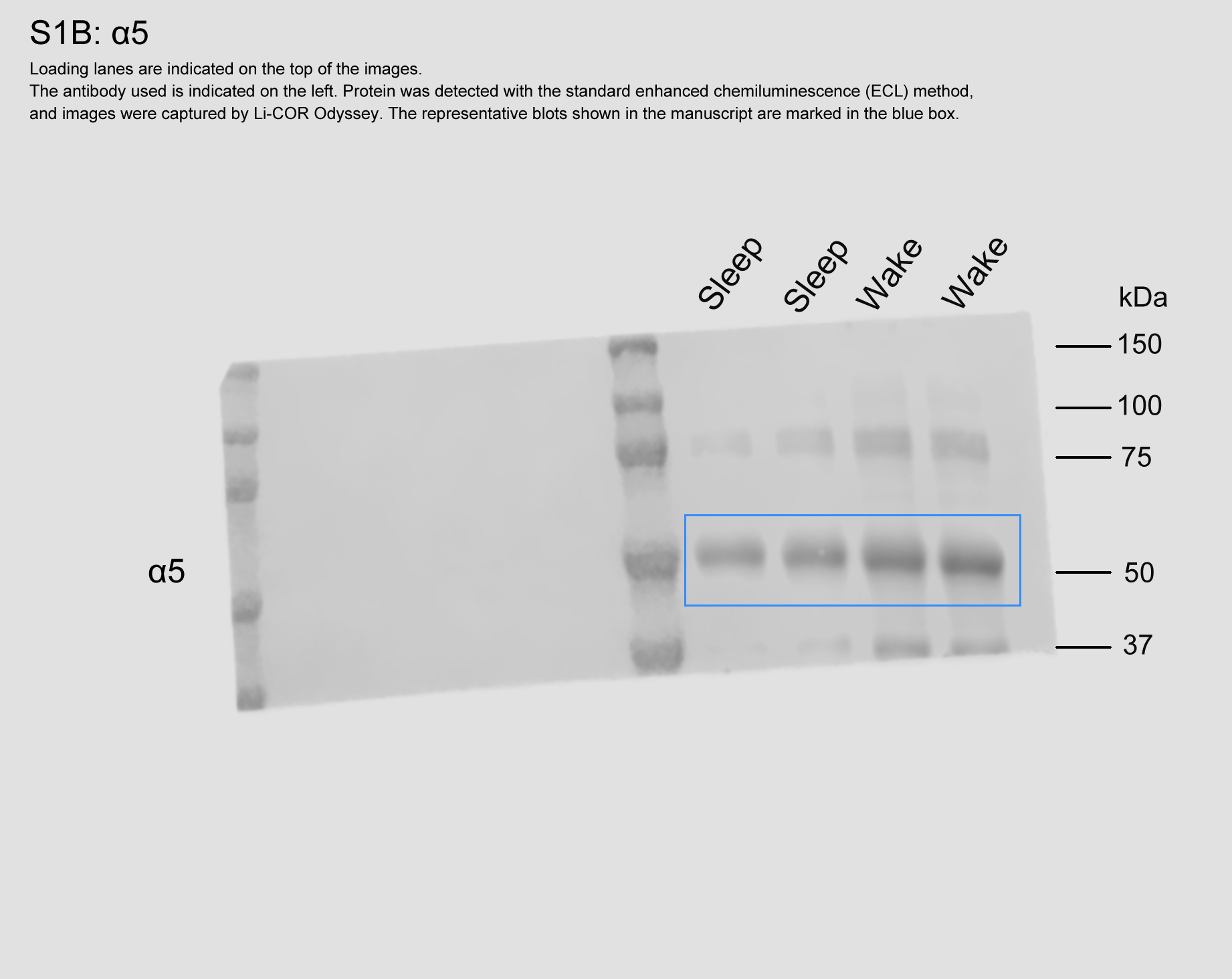

Supplement: S1 Raw Images — (ZIP) [file pbio.3001812.s006.zip › S1_raw_images/S1B/S1B-a5.tif]

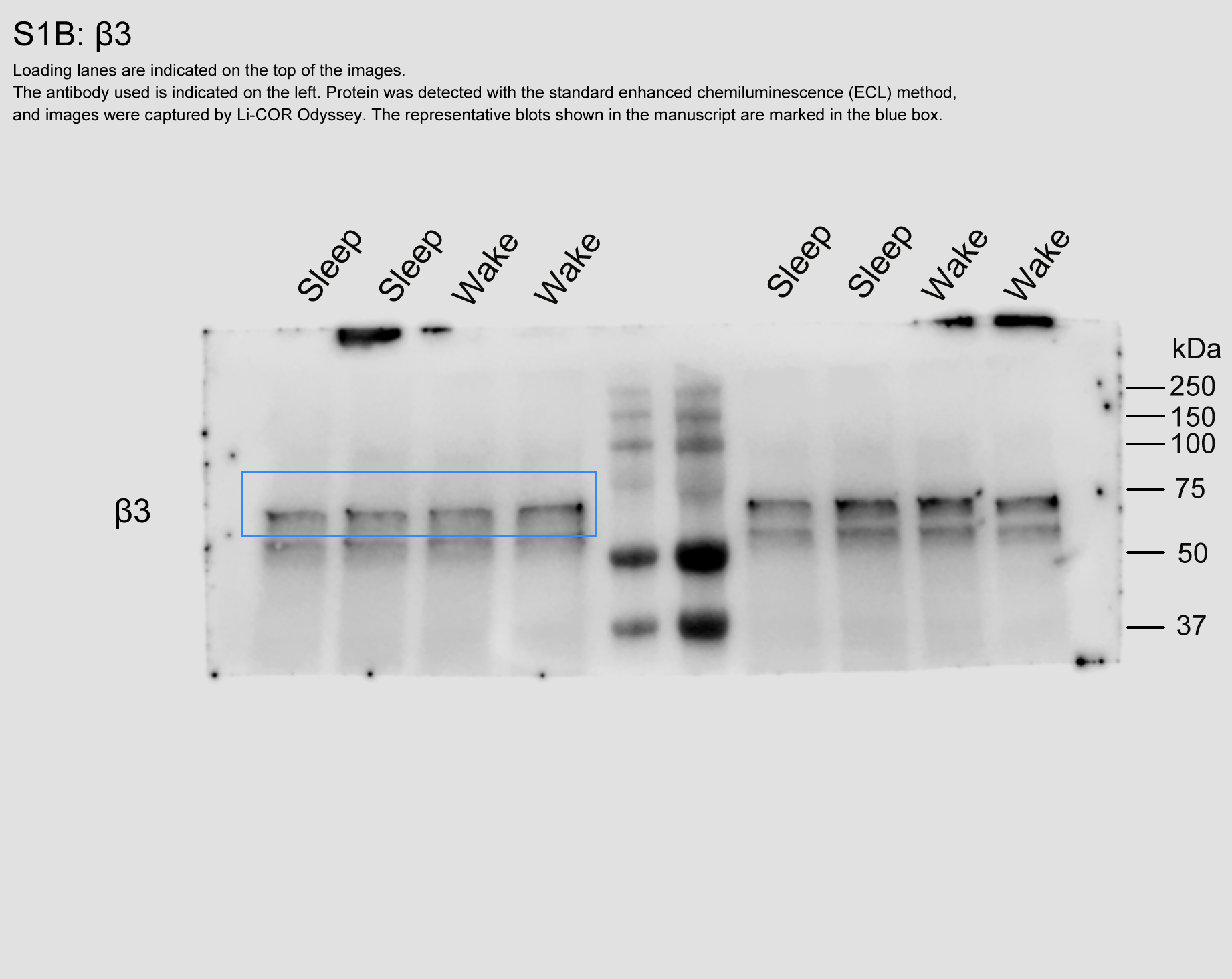

Supplement: S1 Raw Images — (ZIP) [file pbio.3001812.s006.zip › S1_raw_images/S1B/S1B-beta3.tif]

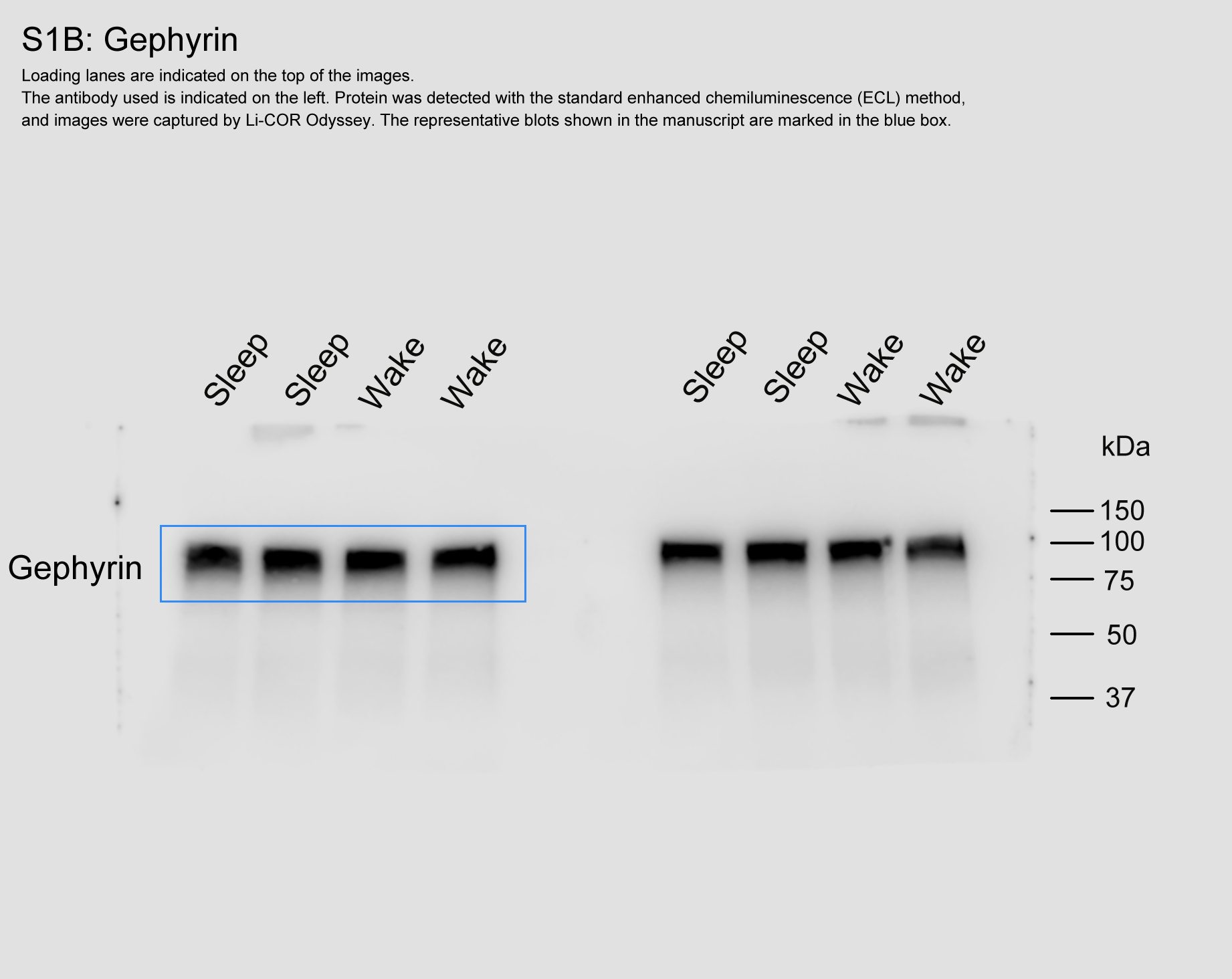

Supplement: S1 Raw Images — (ZIP) [file pbio.3001812.s006.zip › S1_raw_images/S1B/S1B-gephyrin.tif]

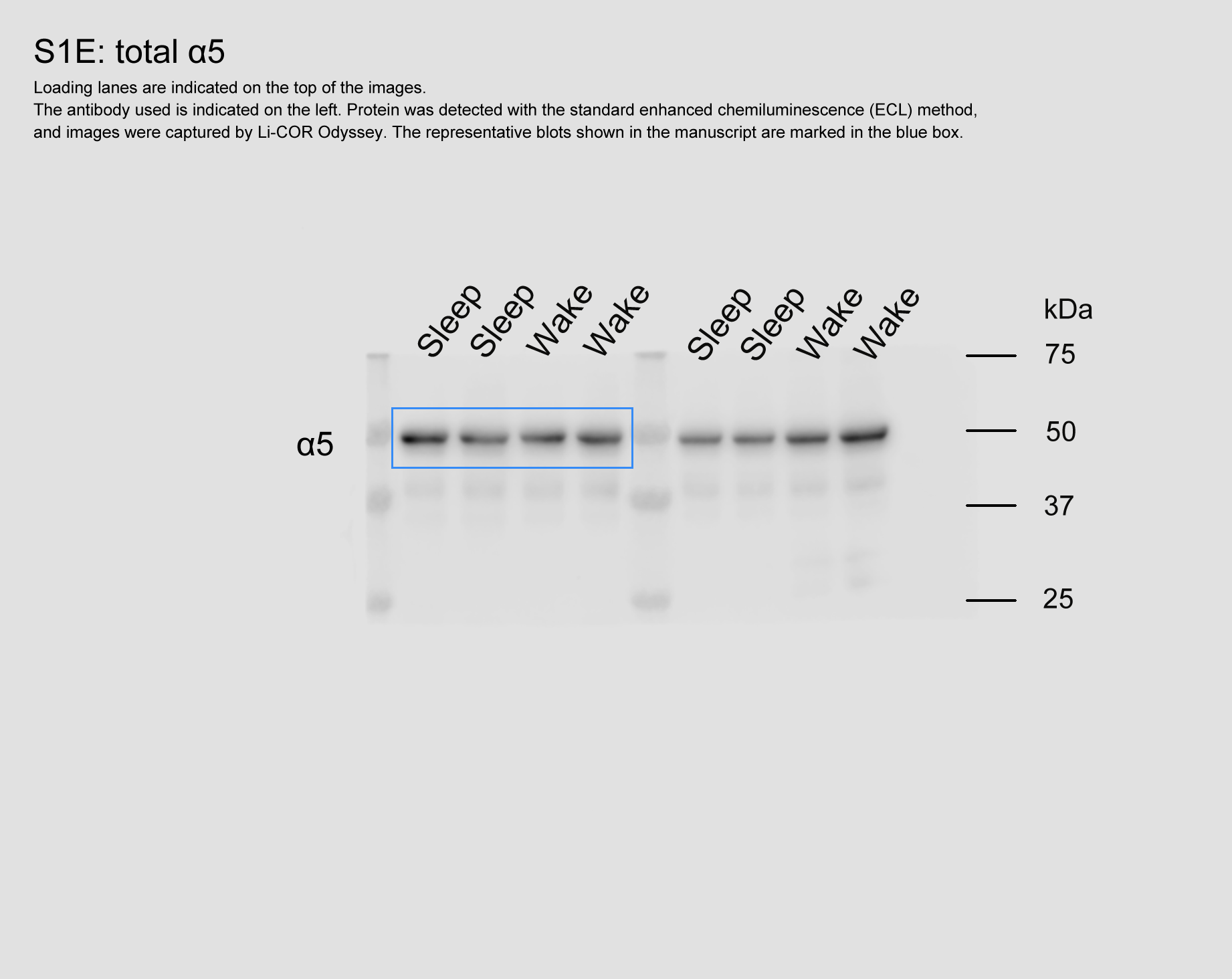

Supplement: S1 Raw Images — (ZIP) [file pbio.3001812.s006.zip › S1_raw_images/S1E/S1E- total a5.tif]

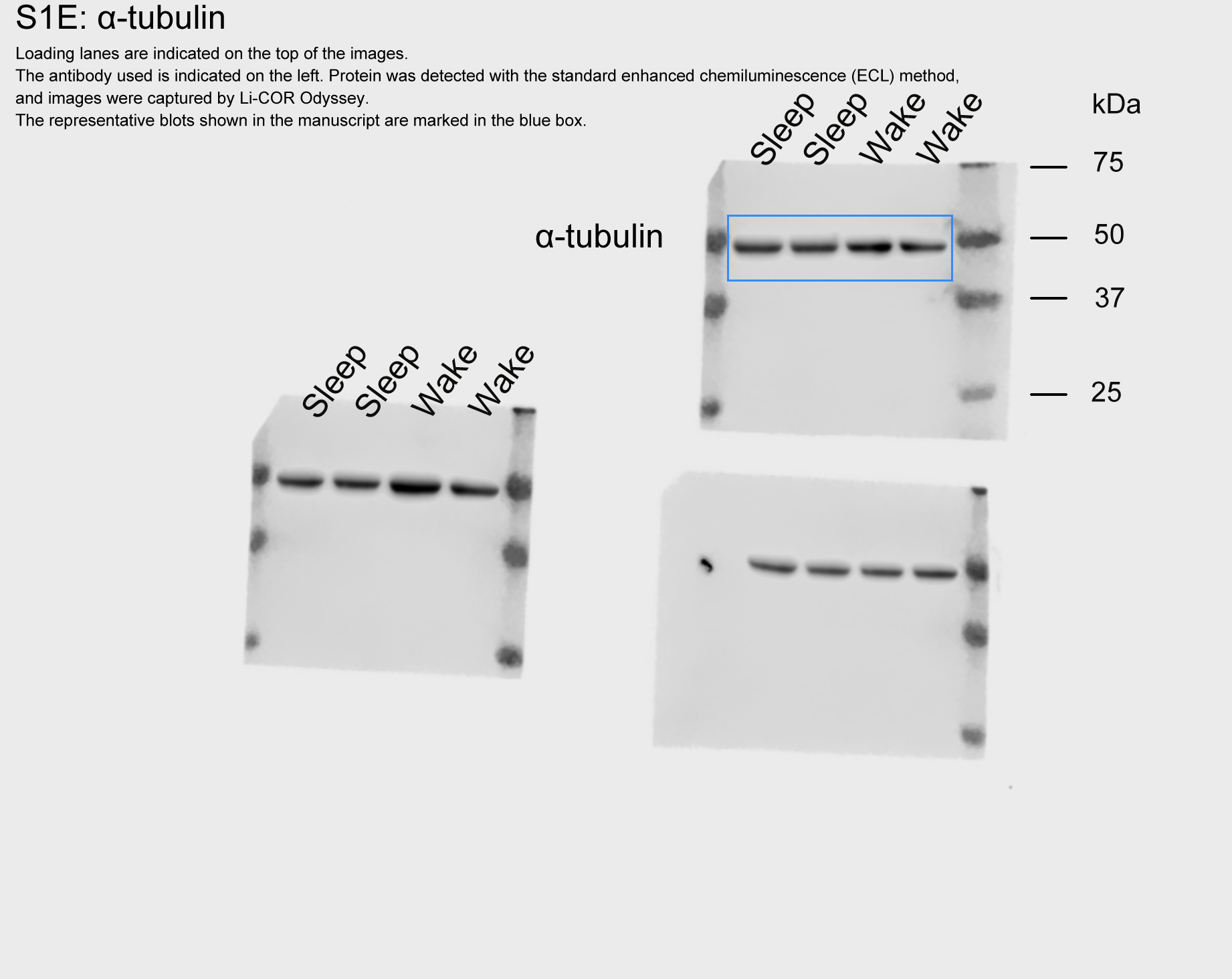

Supplement: S1 Raw Images — (ZIP) [file pbio.3001812.s006.zip › S1_raw_images/S1E/S1E-a-tubulin.tif]

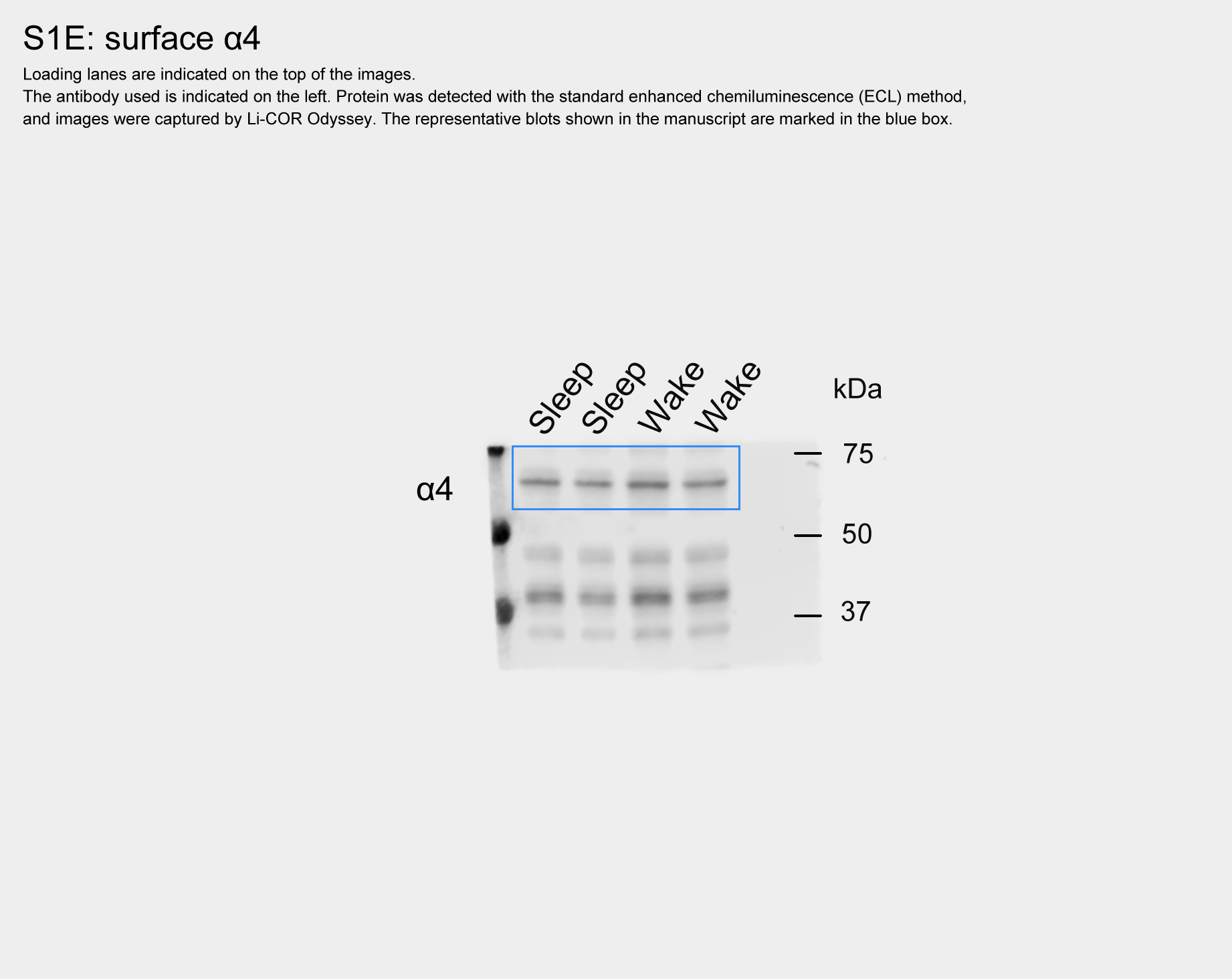

Supplement: S1 Raw Images — (ZIP) [file pbio.3001812.s006.zip › S1_raw_images/S1E/S1E-surface a4.tif]

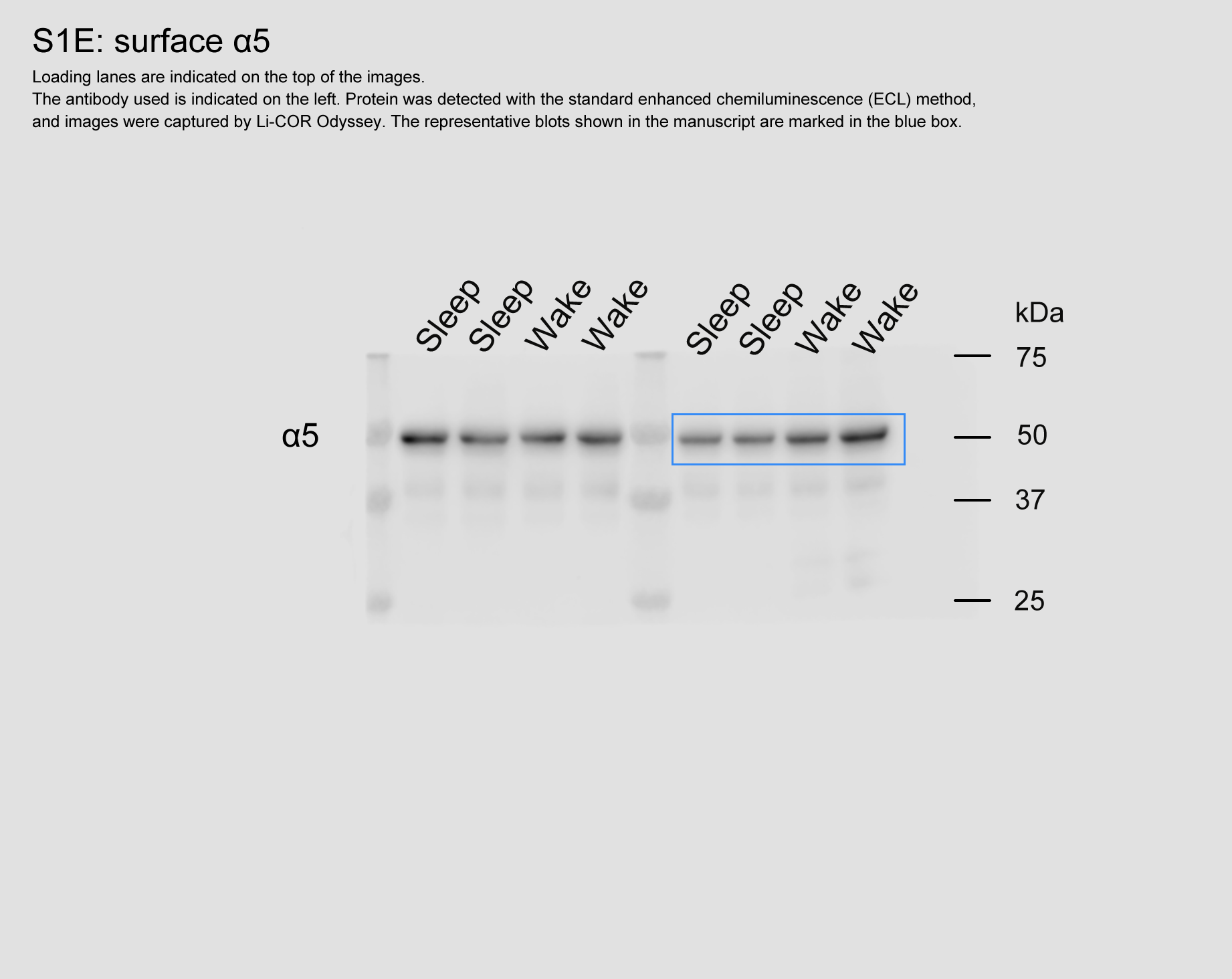

Supplement: S1 Raw Images — (ZIP) [file pbio.3001812.s006.zip › S1_raw_images/S1E/S1E-surface a5.tif]

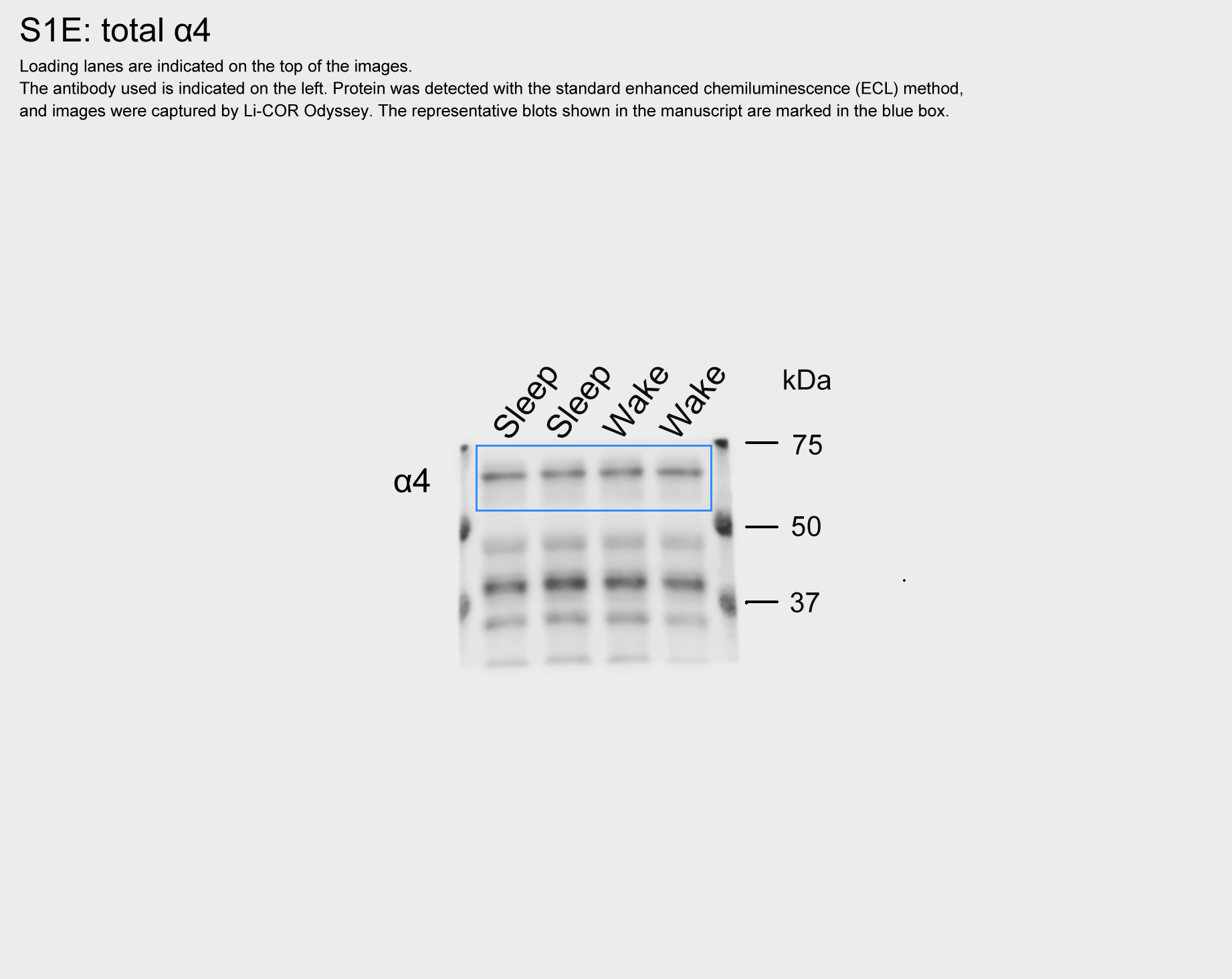

Supplement: S1 Raw Images — (ZIP) [file pbio.3001812.s006.zip › S1_raw_images/S1E/S1E-total a4.tif]

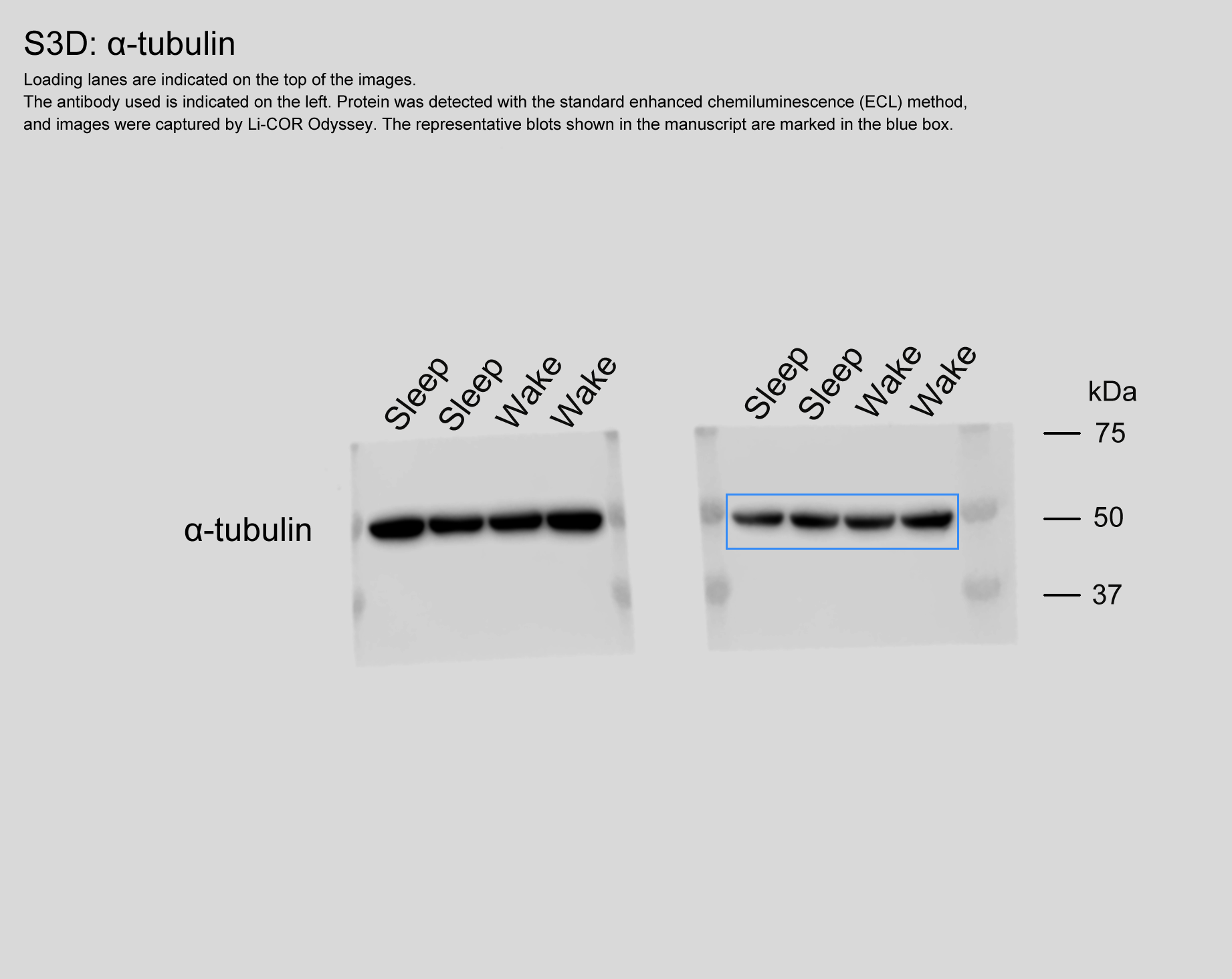

Supplement: S1 Raw Images — (ZIP) [file pbio.3001812.s006.zip › S1_raw_images/S3D/S3D-a-tubulin.tif]

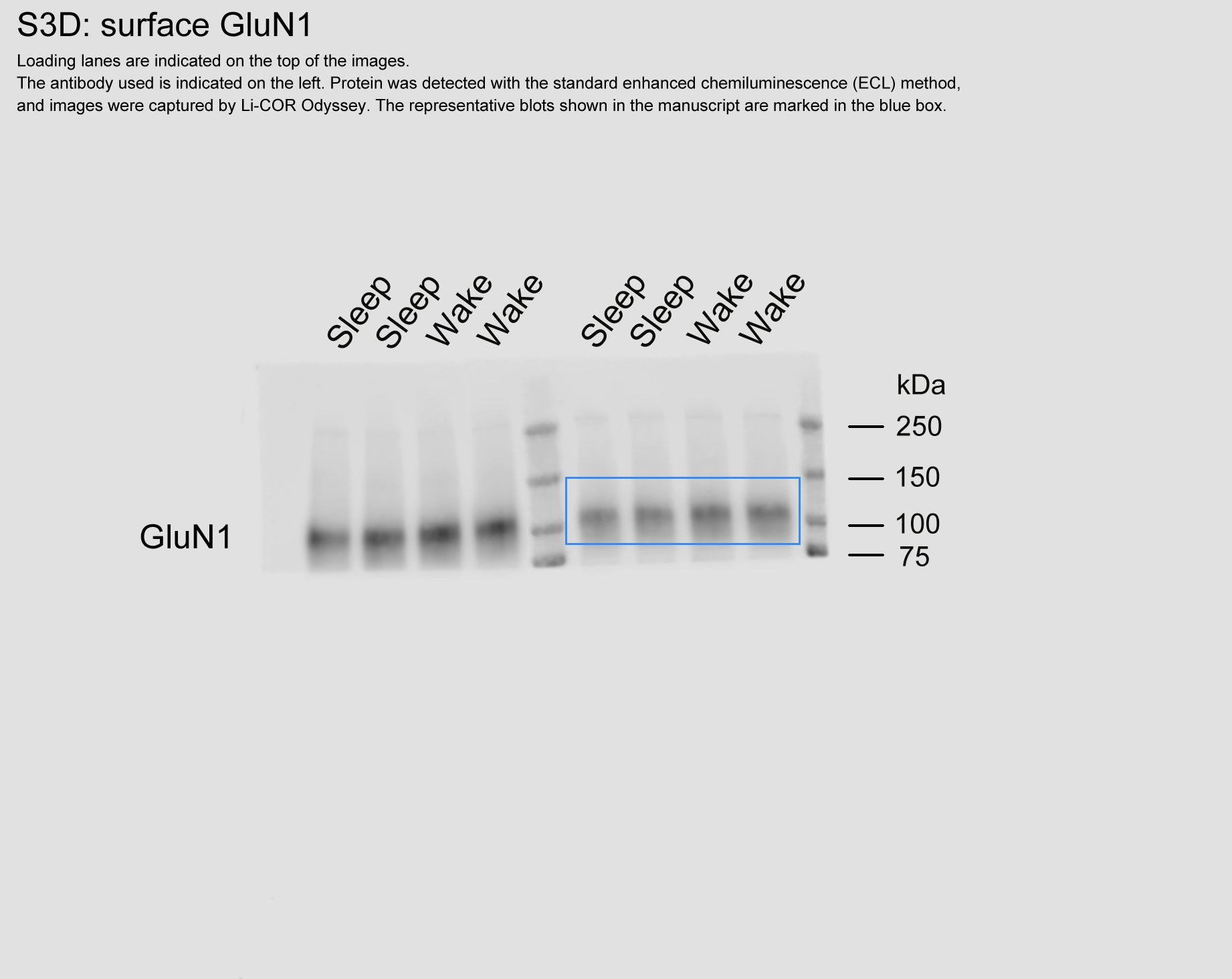

Supplement: S1 Raw Images — (ZIP) [file pbio.3001812.s006.zip › S1_raw_images/S3D/S3D-surface GluN1.tif]

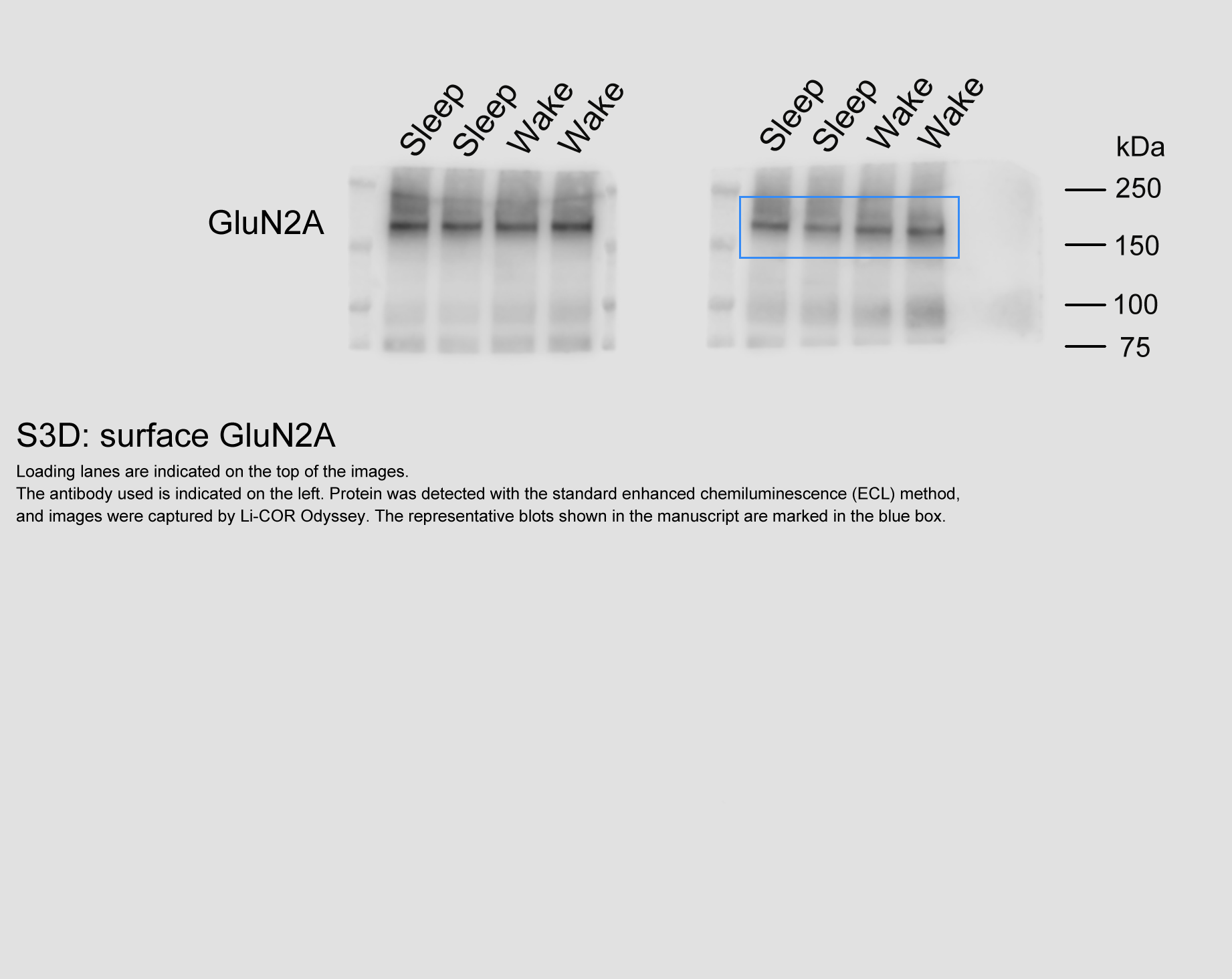

Supplement: S1 Raw Images — (ZIP) [file pbio.3001812.s006.zip › S1_raw_images/S3D/S3D-surface GluN2A.tif]

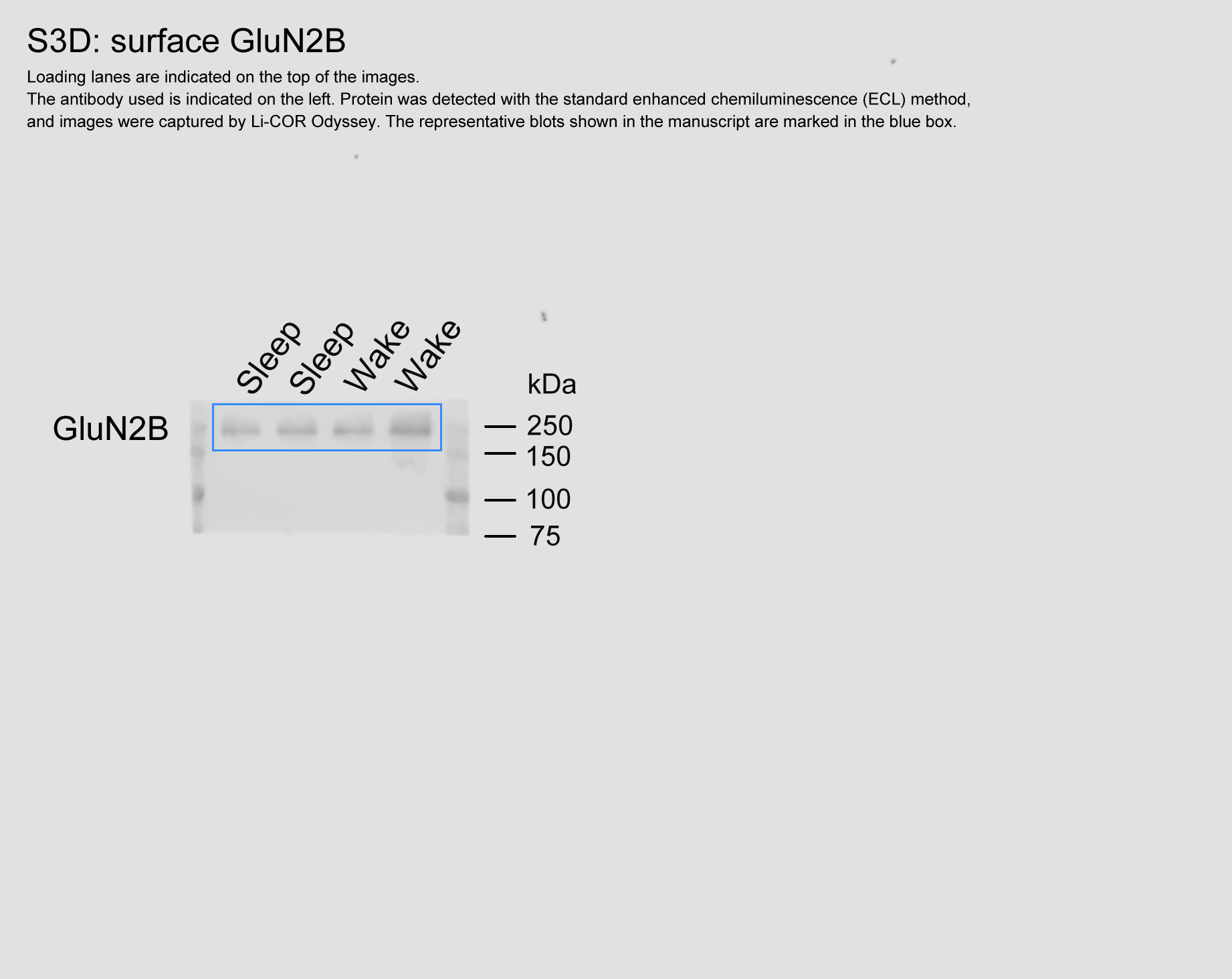

Supplement: S1 Raw Images — (ZIP) [file pbio.3001812.s006.zip › S1_raw_images/S3D/S3D-surface GluN2B.tif]

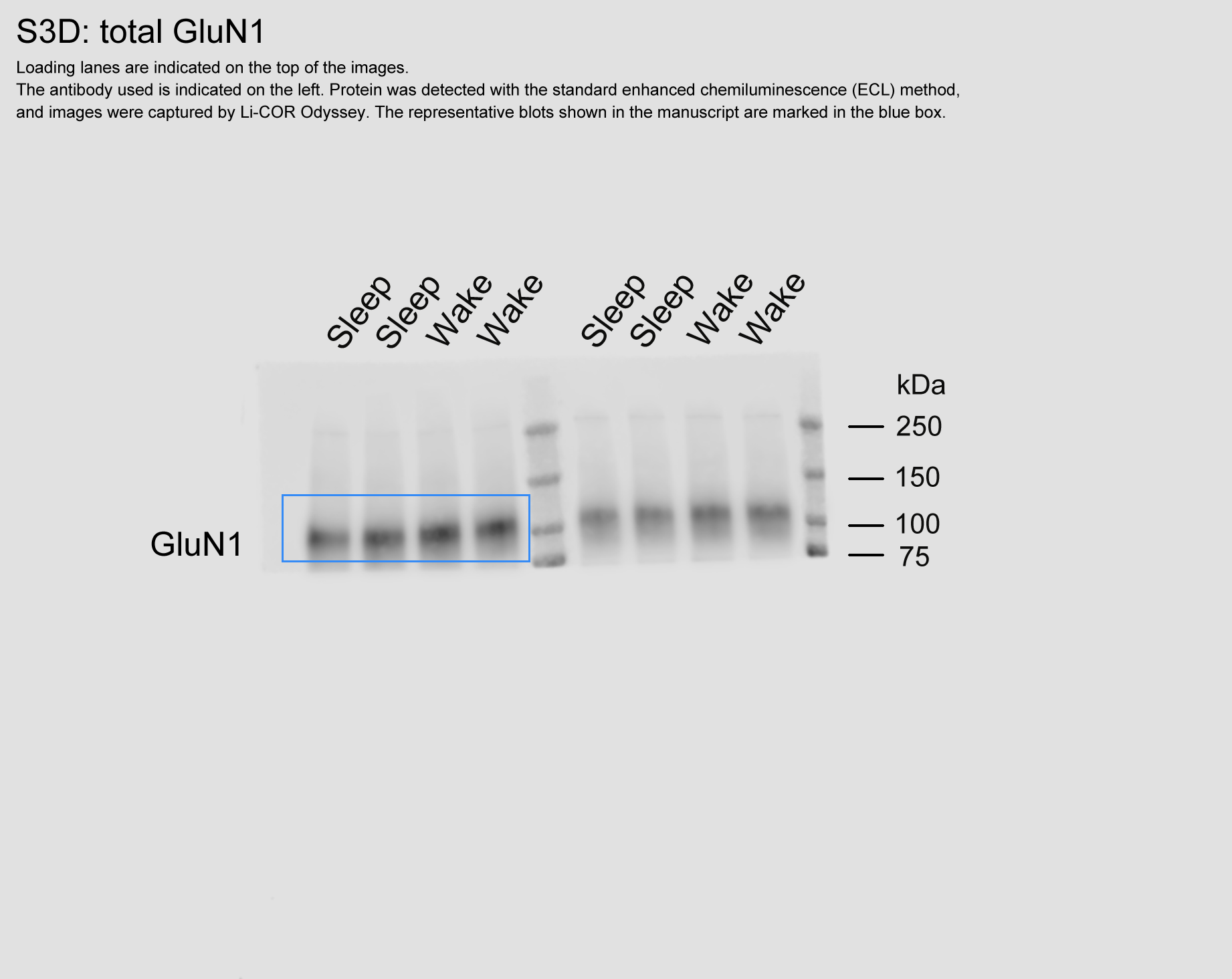

Supplement: S1 Raw Images — (ZIP) [file pbio.3001812.s006.zip › S1_raw_images/S3D/S3D-total GluN1.tif]

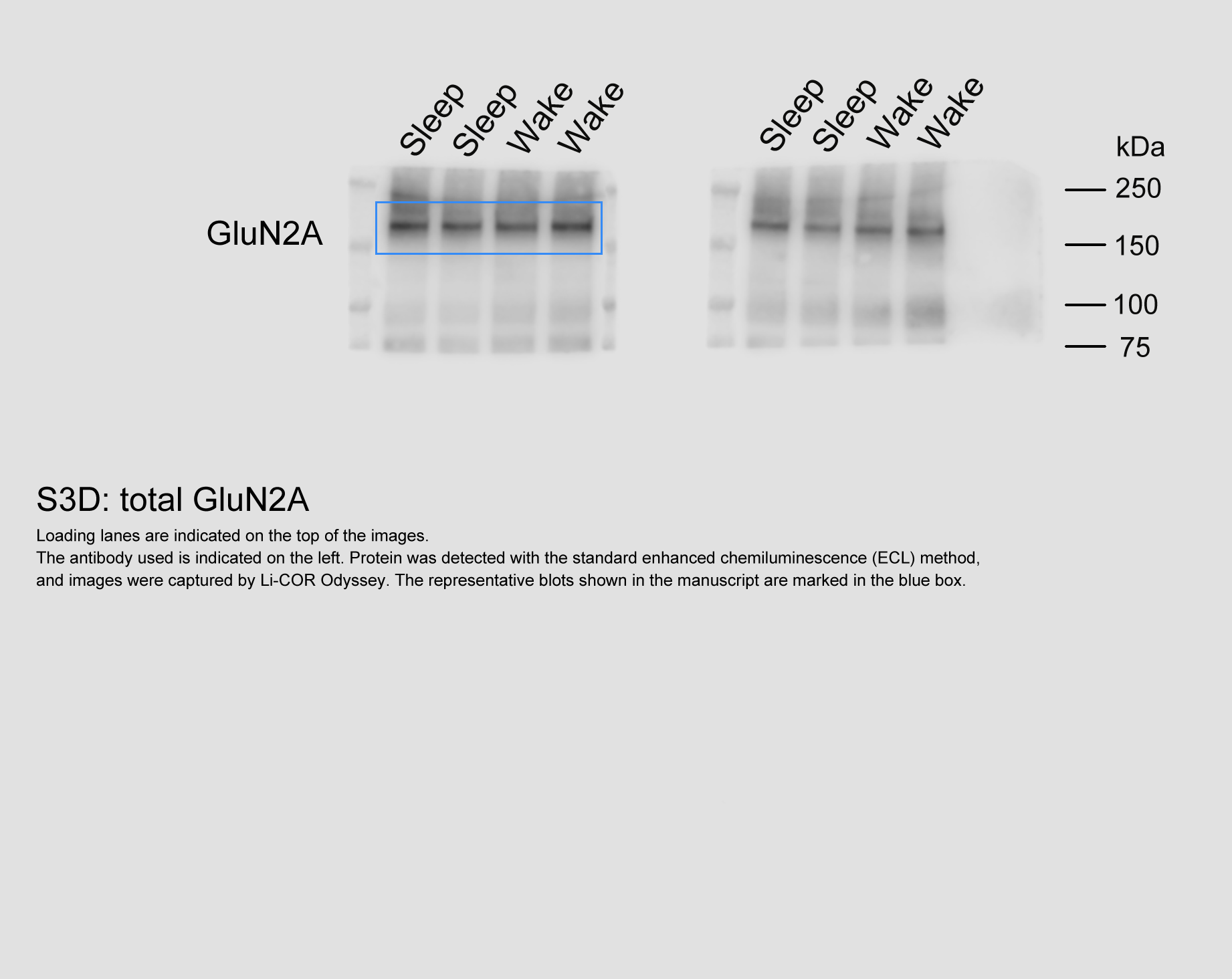

Supplement: S1 Raw Images — (ZIP) [file pbio.3001812.s006.zip › S1_raw_images/S3D/S3D-total GluN2A.tif]

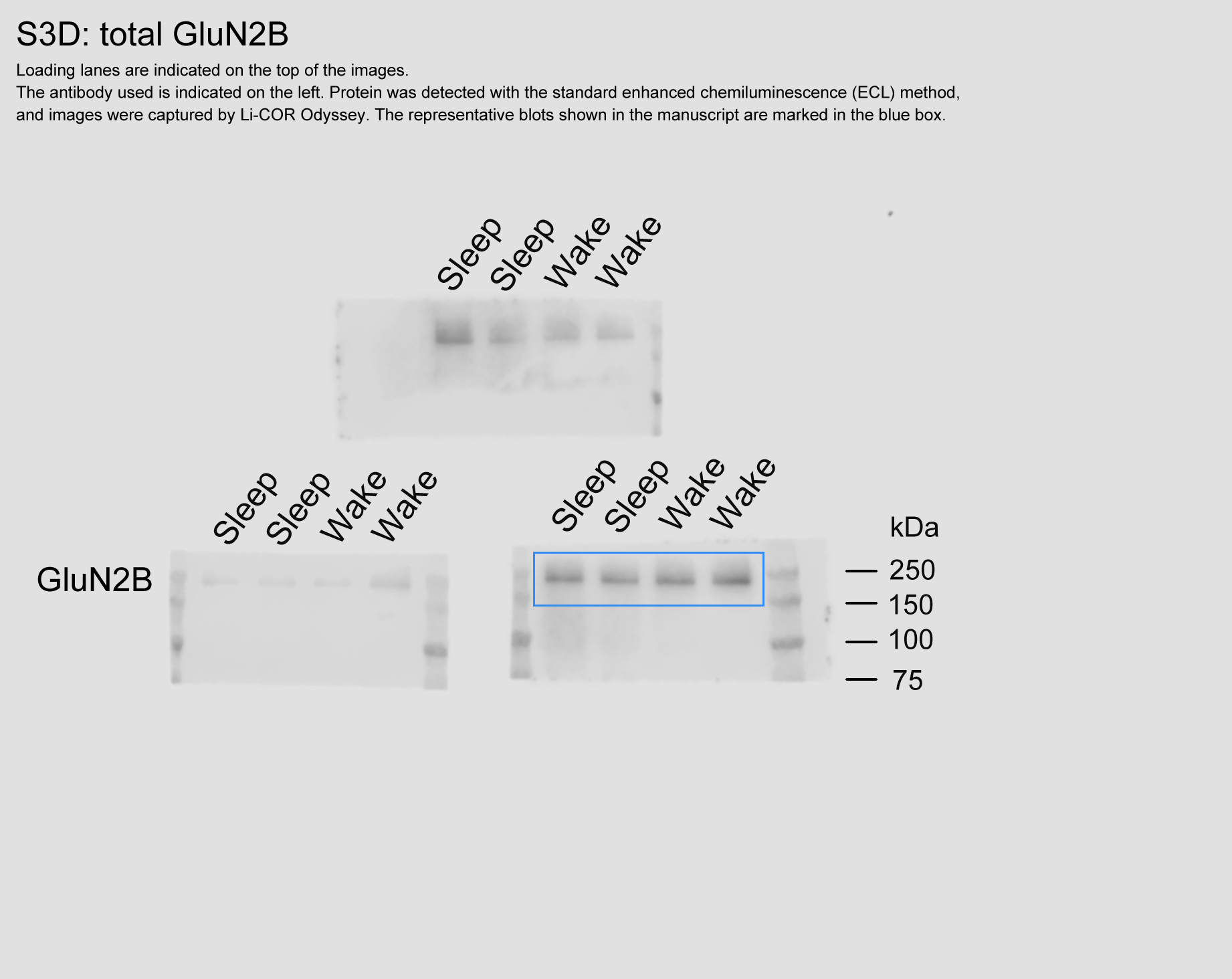

Supplement: S1 Raw Images — (ZIP) [file pbio.3001812.s006.zip › S1_raw_images/S3D/S3D-total GluN2B.tif]
